# Genomically mined acoustic reporter genes for real-time in vivo monitoring of tumors and tumor-homing bacteria

**DOI:** 10.1038/s41587-022-01581-y

**Published:** 2023-01-02

**Authors:** Robert C. Hurt, Marjorie T. Buss, Mengtong Duan, Katie Wong, Mei Yi You, Daniel P. Sawyer, Margaret B. Swift, Przemysław Dutka, Pierina Barturen-Larrea, David R. Mittelstein, Zhiyang Jin, Mohamad H. Abedi, Arash Farhadi, Ramya Deshpande, Mikhail G. Shapiro

**Affiliations:** 1 Division of Biology and Biological Engineering, California Institute of Technology, Pasadena, CA USA; 2 Division of Chemistry and Chemical Engineering, California Institute of Technology, Pasadena, CA USA; 3 Howard Hughes Medical Institute, California Institute of Technology, Pasadena, CA USA; 4 Division of Engineering and Applied Science, California Institute of Technology, Pasadena, CA USA

**Keywords:** Biotechnology, Ultrasound, Synthetic biology

## Abstract

Ultrasound allows imaging at a much greater depth than optical methods, but existing genetically encoded acoustic reporters for in vivo cellular imaging have been limited by poor sensitivity, specificity and in vivo expression. Here we describe two acoustic reporter genes (ARGs)—one for use in bacteria and one for use in mammalian cells—identified through a phylogenetic screen of candidate gas vesicle gene clusters from diverse bacteria and archaea that provide stronger ultrasound contrast, produce non-linear signals distinguishable from background tissue and have stable long-term expression. Compared to their first-generation counterparts, these improved bacterial and mammalian ARGs produce 9-fold and 38-fold stronger non-linear contrast, respectively. Using these new ARGs, we non-invasively imaged in situ tumor colonization and gene expression in tumor-homing therapeutic bacteria, tracked the progression of tumor gene expression and growth in a mouse model of breast cancer, and performed gene-expression-guided needle biopsies of a genetically mosaic tumor, demonstrating non-invasive access to dynamic biological processes at centimeter depth.

## Abstract (web summary)

Gene expression is imaged in deep tissues in mice using ultrasound.

## Main

Basic biological research, in vivo synthetic biology and the development of cell-based medicine require methods to visualize the functions of specific cells deep inside intact organisms. In this context, optical techniques based on fluorescent and luminescent proteins are limited by the scattering and absorption of light by tissue^1^. Ultrasound is a widely used technique for deep tissue imaging, providing sub-100-µm spatial resolution and penetrating several centimeters into tissue^2^. Super-resolution methods^3,4^ have pushed its spatial resolution below 10 µm. Recently, the first genetically encodable reporters for ultrasound^5–7^ were introduced based on gas vesicles (GVs)—air-filled protein nanostructures encoded by clusters of 8–20+ genes, which evolved as flotation devices in diverse, mostly aquatic bacteria and archaea^8,9^. The low density and high compressibility of their air-filled interiors compared to surrounding tissues allow GVs to scatter sound waves and, thereby, produce ultrasound contrast when heterologously expressed as acoustic reporter genes (ARGs) in genetically engineered bacteria^6^ or mammalian cells^7^.

Despite the promise of first-generation ARGs, their utility for monitoring bacterial or mammalian gene expression in vivo is limited. Bacterial ARGs^6^ do not scatter ultrasound non-linearly (making them difficult to distinguish from background tissues), and their in situ expression in vivo is hampered by their poor expression at 37 °C and high metabolic burden. Likewise, mammalian ARGs^7^ produce only linear contrast, and cell-to-cell variability in their expression and burden has limited their use to clonally selected cell lines treated with inhibitors of epigenetic silencing. In both cases, the lack of non-linear signal had to be circumvented by destructive ultrasound imaging pulse sequences, which destroyed the GVs and limited dynamic imaging^10^.

We sought to make acoustic proteins widely useful in in vivo biological research and potential clinical applications by developing ARGs that, when expressed heterologously in either bacteria or mammalian cancer cell lines, could produce GVs with strong non-linear ultrasound contrast and enable long-term expression under physiological conditions. We used a genomic mining approach—previously applied to improving fluorescent proteins^11–14^, opsins^15–17^, Cas proteins^18–22^ and other biotechnology tools^23–28^—to identify ARGs with improved properties, which we subsequently optimized through genetic engineering. By cloning and screening 15 distinct polycistronic operons chosen from a diverse set of 288 GV-expressing species representing a broad phylogeny, we identified two GV gene clusters—from *Serratia sp*. 39006 and *Anabaena flos-aquae*—that produce 9-fold or 38-fold stronger non-linear acoustic contrast than previously tested clusters when expressed in bacteria and mammalian cells, respectively. The bacterial ARG adapted from *Serratia* sp. 39006 (bARG_Ser_), when expressed in the probiotic bacterium *Escherichia coli* Nissle 1917 (EcN), enabled non-invasive ultrasound imaging of these bacteria colonizing tumors at depths greater than 1 cm, providing direct visualization of the microscale in vivo distribution of this potential anti-cancer therapy^29–32^. The mammalian ARG adapted from *A. flos-aquae* (mARG_Ana_), when expressed in human breast cancer cells, enabled both the non-invasive, in situ microscale imaging and long-term monitoring of heterologous gene expression in developing orthotopic tumors and the ultrasound-guided biopsy of a genetically defined subpopulation of these tumor cells. The properties and performance of these improved ARGs should facilitate a wide range of in vivo research.

## Results

### Genomically mined GV gene clusters with improved ultrasound contrast

**Fig. 1:**
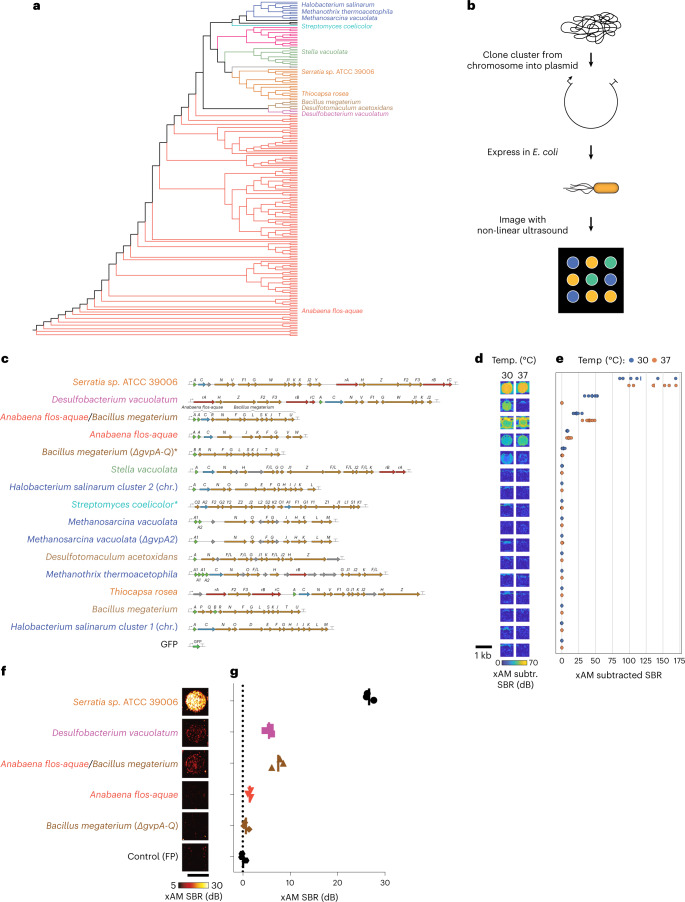
Genomic mining of GV gene clusters reveals homologs with non-linear ultrasound contrast in *E. coli*. **a**, 16S phylogenic tree of known GV-producing organisms, with the species from which GV genes were cloned and tested in this study indicated by name. See Supplementary Fig. 1 for the fully annotated phylogenic tree. *B. megaterium* and *S. coelicolor* were not reported to produce GVs, but we tested their GV gene clusters here based on previous expression of *B. megaterium* GVs in in *E. coli*^38^ and to broadly sample the phylogenetic space by including an actinomycete. **b**, Workflow for testing GV clusters. Selected GV gene clusters were expressed in BL21(DE3) *E. coli* by growing patches of cells on plates containing the inducer IPTG, and the patches were then imaged with non-linear ultrasound (xAM). **c**–**e**, Diagrams of the GV gene clusters tested in *E. coli* (**c**), differential xAM images of representative patches (**d**) and quantification of the differential xAM signal-to-background ratio (SBR) of the patches (*n* = 6 biological replicates) (**e**). **f**,**g**, Representative xAM images (**f**) and quantification of the xAM SBR (*n* = 3 biological replicates, each with two technical replicates; lines represent the mean) (**g**) for the top five GV-producing clusters expressed in *E. coli* at 30 °C on solid media and normalized to 5 × 10^9^ cells per milliliter in agarose phantoms, imaged at 1.74 MPa. See Extended Data Fig. 2a,b for the ultrasound signal at varying acoustic pressures and Extended Data Fig. 2c for the corresponding BURST data. Source data

GVs are encoded by polycistronic gene clusters comprising one or more copies of the primary structural gene *gvpA* and 7–20+ other genes encoding minor constituents, assembly factors or reinforcing proteins, which together help assemble the GVs’ protein shells^9^. Starting with a list of organisms with confirmed GV production and sequenced operons (Supplementary Table 1), we cloned GV operons from 11 representative species, providing a broad sampling of phylogenetic space, cluster architecture and organismal characteristics (that is, halophilic, thermophilic and mesophilic) (Fig. 1a and Supplementary Fig. 1).

**Extended Data Fig. 1:**
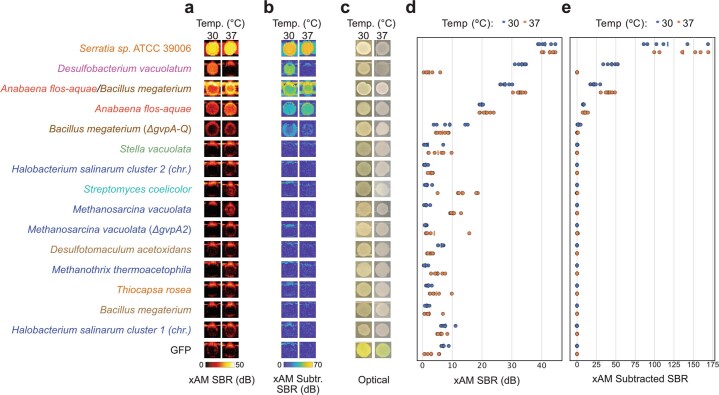
Additional images and quantification of *E. coli* patches expressing select GV gene clusters. **(a-c)** xAM images (a), pre-minus-post-collapse xAM images (b), and optical images showing opacity (c) of patches of *E. coli* expressing various GV gene clusters from the organisms listed on the left. (**d-e**) Quantification of the xAM (d) and pre-minus-post-collapse xAM (e) signals from images in (a-b) (n = 6 biologically independent samples examined in one experiment). SBR, signal-to-background ratio. Source data

We expressed each operon in confluent *E. coli* patches at several temperatures and inducer concentrations (Fig. 1b), comparing them to two bacterial ARG constructs previously shown to work in *E. coli*^6^—bARG1 (*Anabaena flos-aquae* (NCBI: txid315271)*/Bacillus megaterium* (NCBI: txid1404) hybrid) and *Bacillus megaterium ΔgvpA-Q*—as well as the full *Bacillus megaterium* gene cluster (Fig. 1c–g, Extended Data Fig. 1a–e and Supplementary Figs. 2a–c and 3–5). We scanned these patches using a home-built robotic ultrasound imaging apparatus, applying a cross-propagating amplitude modulation pulse sequence (xAM)^33^. This pulse sequence enhances signals specific to non-linear contrast agents, such as GVs, while canceling linear background scattering. Unlike pulse sequences that rely on the irreversible collapse of GVs to obtain GV-specific contrast^6,7,10^, xAM is non-destructive. In addition, we examined the optical opacity of the patches, which can be increased by sufficient levels of GV expression.

Of the 15 gene clusters tested, only three showed substantial xAM signal when expressed at 37 °C, and five showed substantial xAM signal at 30 °C (Fig. 1c–e). Even though all operons tested are from organisms reported to produce GVs in their native hosts, only the *A. flos-aquae*, *B. megaterium ΔgvpA-Q*, bARG1, *Desulfobacterium vacuolatum* (NCBI: txid1121400) and *Serratia sp*. 39006 (*Serratia*; NCBI: txid104623) clusters produced detectable GVs when expressed heterologously in *E. coli*. Several other operons produced a small amount of ultrasound contrast under certain conditions, which did not arise from GV expression but reflected a rough patch morphology likely due to cellular toxicity (Supplementary Fig. 2d). The failure of most tested gene clusters to produce GVs in *E. coli* is not surprising given the complexity of polycistronic heterologous expression, which requires each component protein to fold and function properly in a new host with a potentially different cytoplasmic environment, growth temperature and turgor pressure^34,35^. In addition, it is possible that some genes included in the clusters act as *cis*-regulators^9,34,36–38^, limiting expression absent a specific *trans* input, or that some additional genes are required beyond the annotated operons.

**Extended Data Fig. 2:**
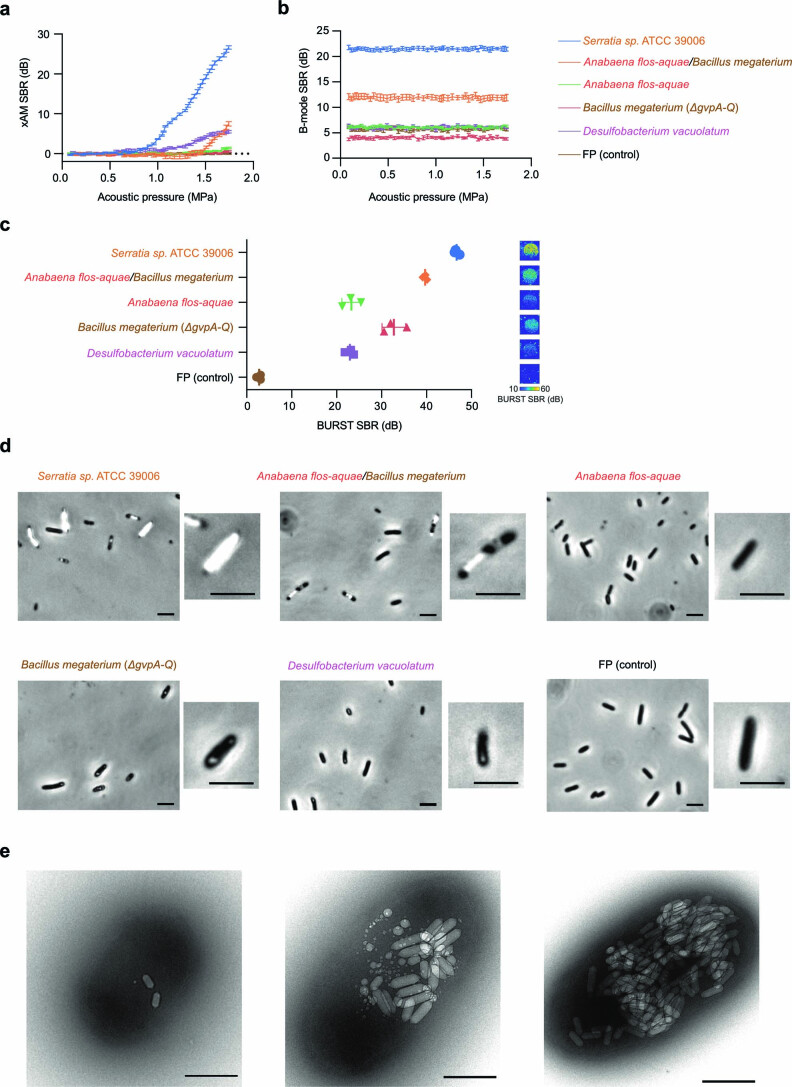
Characterization of working GV clusters in BL21(DE3) *E. coli*. (**a-d**) xAM signal-to-background ratio (SBR) as a function of acoustic pressure (a), B-mode SBR at a constant pressure of 0.15 MPa after each increase in acoustic pressure in a (b), BURST SBRs and corresponding representative images (c), and representative phase contrast microscopy (PCM) images (from at least n = 3 biological replicates) (d) of the working GV clusters expressed in BL21(DE3) *E. coli* at 30 °C on solid media. For ultrasound imaging (a-c), samples were normalized to 5 × 10^9^ cells/mL in agarose phantoms. Curves and error bars represent the mean (n = 3 biological replicates each with 2 technical replicates) ± SD. (a-b) have the same legend. GV clusters in cells are visible by PCM for all clusters except for the cluster from *Anabaena flos-aquae* and the fluorescent protein (FP) control (d). (**e**) Representative TEM images of BL21(DE3) *E. coli* cells expressing Serratia GVs at varying levels of expression (from n = 17 images from a single sample). Scale bars are 5 μm in (d) and 500 nm in (e). Source data

In patch format, the strongest acoustic performance was observed with the genes from *Serratia*, bARG1, *A. flos-aquae*, *B. megateriaum* and *D. vacuolatum*. To compare these clusters while controlling for cell density, we resuspended cells expressing them in hydrogels at equal densities and imaged them using both xAM (Fig. 1f,g and Extended Data Fig. 2a,b) and a more sensitive but destructive method called BURST^10^ (Extended Data Fig. 2c) and examined them optically with phase-contrast microscopy (PCM), which reveals the presence of GVs due to the refractive index difference between GVs and water^39,40^ (Extended Data Fig. 2d). Three of the clusters produced xAM signals, and all clusters produced BURST signals significantly stronger than the negative control. All clusters except *A. flos-aquae* exhibited sufficient GV expression to be visible by PCM.

Cells expressing the *Serratia* cluster produced the strongest ultrasound signals, 19.2 dB above the next brightest cluster, bARG1, under xAM imaging at an applied acoustic pressure of 1.74 MPa: a 9-fold gain in signal intensity (Fig. 1f). Additionally, PCM and transmission electron microscopy (TEM) images showed that cells expressing the *Serratia* cluster had the highest levels of GV expression (Extended Data Fig. 2d,e).

Because overexpression of any protein imposes a metabolic demand on the host cell^41–43^, we reasoned that deletion of non-essential genes could improve GV expression from the *Serratia* cluster and, therefore, the xAM signal. Previous work showed that deletions of *gvpC*, *gvpW*, *gvpX*, *gvpY, gvpH* or *gvpZ* preserve GV formation in the native organism^34^. We tested these deletions as well as the deletion of an unannotated hypothetical protein (Ser39006_001280) (Supplementary Fig. 6a). When expressed in *E. coli*, deletions of *gvpC*, *gvpH*, *gvpW*, *gvpY* or *gvpZ* reduced or eliminated xAM signal (Supplementary Fig. 6b,c) and patch opacity (Supplementary Fig. 6d). Deletion of *gvpX* increased xAM signal but decreased opacity, and deletion of Ser39006_001280 increased both xAM signal and opacity. Based on these results, we selected the *Serratia* ΔSer39006_001280 operon for subsequent in vitro and in vivo experiments. We call this genetic construct bARG_Ser_—a bacterial acoustic reporter gene derived from *Serratia*.

### bARG_Ser_ shows robust performance in EcN

**Fig. 2:**
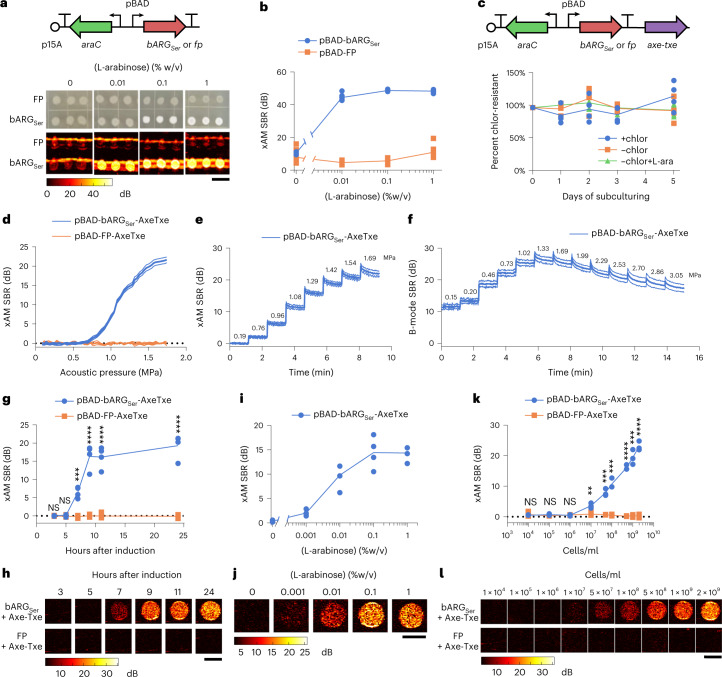
Expression of bARG_Ser_ in EcN and acoustic characterization in vitro. **a**, Diagram of the arabinose-inducible construct pBAD-bARG_Ser_ used to express bARG_Ser_ in EcN (top) and optical and xAM images of bARG_Ser_-expressing or FP-expressing patches of EcN on solid media with varying L-arabinose concentrations (bottom). Scale bar, 1 cm. See Extended Data Fig. 3 for corresponding results with IPTG-inducible and aTc-inducible constructs. **b**, Quantification of xAM signal-to-background ratio (SBR) of patches from **a** versus the L-arabinose concentration (*n* = 8). **c**, Diagram of the construct from **a** with Axe-Txe^47^ added, creating pBAD-bARG_Ser_-AxeTxe, to enable plasmid maintenance in the absence of antibiotics (top) and verification of plasmid maintenance (bottom). Conditions were with chloramphenicol (+chlor), without chloramphenicol (−chlor) or without chloramphenicol and with 0.1% L-arabinose (−chlor +L-ara) using pBAD-bARG_Ser_-AxeTxe EcN (*n* = 4). **d**, xAM SBR as a function of transmitted acoustic pressure. **e**,**f**, xAM (**e**) and parabolic B-mode (**f**) SBRs measured over time when the transmitted acoustic pressure was increased every ~70 seconds, and the pulse repetition rate was 86.8 Hz. For **d**–**f**, cells were induced with 0.1% L-arabinose for 24 hours. Bold lines represent the mean, and thin lines represent ± standard deviation (*n* = 3 biological replicates, each with two technical replicates). **g**,**h**, xAM ultrasound SBR (**g**) and corresponding representative images (*P* values: 0.519494, 0.240386, 0.000120555, 6.818737 × 10^−5^, 3.683585 × 10^−5^ and 2.325819 × 10^−5^) (**h**) at several timepoints after inducing with 0.1% L-arabinose. **i**,**j**, xAM SBR (**i**) and corresponding representative images (**j**) after inducing with varying L-arabinose concentrations for 24 hours. **k**,**l**, xAM SBR (*P* values: 0.699456, 0.0568424, 0.597418, 0.00906739, 0.00046697, 0.000456979, 4.937128 × 10^−6^, 0.000183889 and 1.708183 × 10^−5^) (**k**) and corresponding representative images (**l**) of varying concentrations of cells induced for 24 hours with 0.1% L-arabinose in liquid culture. For **h**,**j**,**l**, scale bars are 2 mm. For **d**–**j**, cells were grown in liquid culture and normalized to 10^9^ cells per milliliter for ultrasound imaging. For **g**,**i**,**k**, each point is a biological replicate (*n* = 4 for **g** and **i**; *n* = 3 for **k**) that is the average of at least two technical replicates. Curves represent the mean for **b**–**k**. Asterisks represent statistical significance by two-tailed, unpaired Student’s *t*-tests (****P < 0.0001; ***P < 0.001; **P < 0.01; NS, not significant). Source data

**Extended Data Fig. 3:**
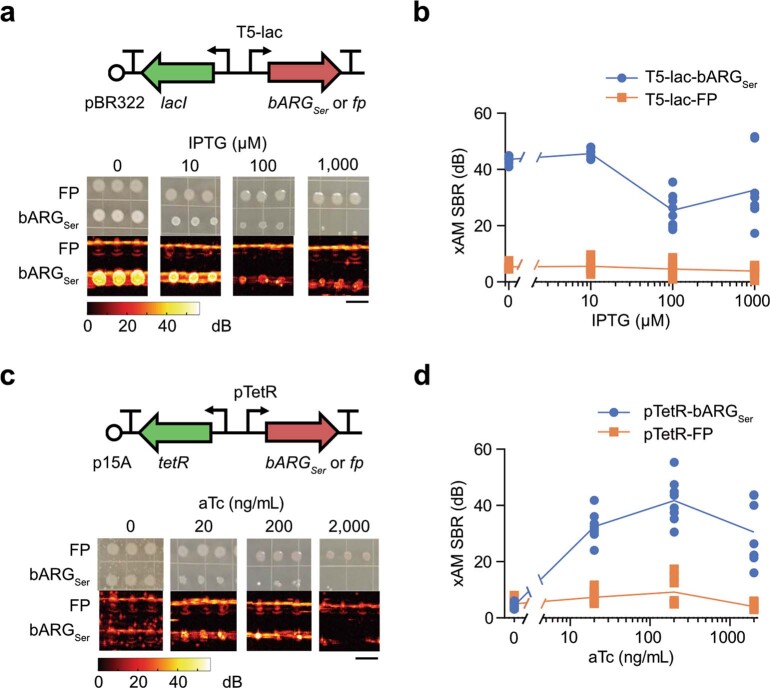
Testing bARG_Ser_ expression in EcN with IPTG- and aTc-inducible gene circuits. (**a**) Diagram of the IPTG-inducible construct used to express bARG_Ser_ in EcN (top), and representative optical and xAM images of bARG_Ser_-expressing or fluorescent protein (FP)-expressing patches of EcN on solid media with varying IPTG concentrations at 37 °C (bottom). (**b**) Quantification of the xAM SBR of all patches from the experiment in (a) (n = 8 biologically independent samples examined in one experiment; curves represent the mean). (**c-d**) Same as in (a-b) but for the aTc-inducible construct. The scale bars in (a,c) represent 1 cm. Source data

We transferred bARG_Ser_ into EcN, a strain of *E. coli* that is widely used in in vivo biotechnology applications due to its ability to persist in the gastrointestinal (GI) tract and colonize tumors^44–46^ and to deliver anti-tumor therapy^29–32^. We tested three different inducible promoter architectures and found that the L-arabinose-inducible pBAD promoter provided the most robust control over GV expression without obvious burden at 37 °C (Fig. 2a,b and Extended Data Fig. 3). To enable the pBAD-bARG_Ser_ plasmid to be maintained without antibiotic selection, as required in certain in vivo applications, we added the toxin–antitoxin stability cassette Axe-Txe^47^. This allowed the plasmid to be maintained in EcN for up to 5 days of daily subculturing in liquid media without antibiotics, both with and without ARG induction (Fig. 2c).

**Extended Data Fig. 4:**
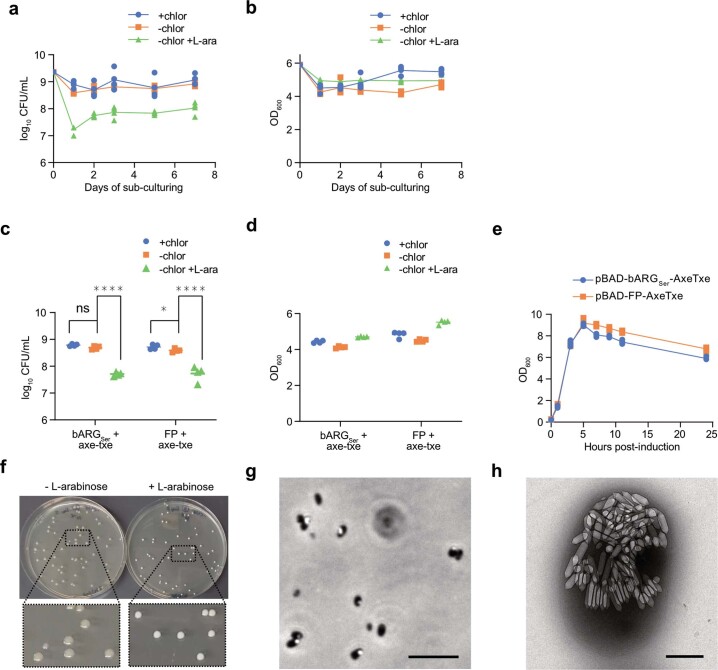
Effect of induction on viability and OD_600_ for bARG_Ser_-expressing EcN in liquid culture. (**a-b**) Colony forming units (CFU) per mL of culture (a) and optical density at 600 nm (b) during daily sub-culturing into LB media with 25 μg/mL chloramphenicol (+chlor), without chloramphenicol (-chlor), or without chloramphenicol and with 0.1% (w/v) L-arabinose (-chlor +L-ara) using pBAD-bARG_Ser_-AxeTxe EcN. Curves represent the mean of n = 4 biological replicates. (**c-d**) Colony forming units (CFUs) per mL of culture on chloramphenicol plates (c) and optical density at 600 nm (p values from left to right: 0.0638556, 1.019913e-6, 0.0217493, 3.800448e-6) (d) of pBAD-bARG_Ser_-AxeTxe EcN and pBAD-FP-AxeTxe EcN cultures 24 hours after sub-culturing into LB media with the same conditions as in (a-b) and in Fig. 2c. Asterisks indicate statistical significance by two-tailed, unpaired Student’s t-tests (**** = p < 0.0001, * = p < 0.05, ns = no significance); n = 4 biological replicates. (**e**) OD_600_ versus time after inducing pBAD-bARG_Ser_-AxeTxe and pBAD-FP-AxeTxe EcN strains with 0.1% (w/v) L-arabinose in liquid culture at 37 °C (n = 4 biological replicates). Between 5 and 24 hours post-induction, when the OD_600_ of all cultures decreased, the OD_600_ of FP-expressing cultures was slightly higher than that of the bARG_Ser_-expressing cultures, likely due to expression of red fluorescent protein which is known to absorb light at 600 nm^86^. (**f**) Representative image of colonies from the experiment in Fig. 2c on chloramphenicol plates with (right) and without (left) 0.1% L-arabinose. The opacity of the colonies on plates with L-arabinose indicates bARG_Ser_ expression and was used to screen for mutants deficient in bARG_Ser_ expression (see Supplementary Fig. 7). (**g-h**) Representative phase contrast microscopy (from n = 4 biological replicates) (g) and transmission electron microscopy (from n = 23 images taken of a single sample) (h) images of pBAD-bARG_Ser_-AxeTxe EcN cells grown on plates with 0.1% (w/v) L-arabinose at 37 °C. Scale bars are 10 µm (g) and 500 nm (h). Curves and lines represent the mean for a-e. Source data

The expression of most heterologous genes, including widely used fluorescent proteins (FPs), results in some degree of metabolic burden on engineered cells^42,43,48^. Consistent with this expectation, the induction of pBAD-bARG_Ser_ EcN resulted in reduced colony-forming unit counts to an extent similar to the expression of an FP (Extended Data Fig. 4a,c), while culture optical density (OD) remained relatively unchanged (Extended Data Fig. 4b,d). The growth curves of induced bARG_Ser_-expressing and FP-expressing EcN were indistinguishable during the log phase (0–5 hours), indicating that the two strains have similar growth rates (Extended Data Fig. 4e). Collectively, these results suggest that pBAD-driven expression of bARG_Ser_ in EcN is not more burdensome than that of FPs, which are widely accepted as relatively non-perturbative indicators of cellular function.

To further examine the genetic stability of bARG_Ser_ constructs, we plated cells from daily subcultures onto agar with 0.1% (w/v) L-arabinose and examined colony opacity (Extended Data Fig. 4f) as a measure of retained GV expression. Of a total of 3,824 colonies, nearly all were opaque (Extended Data Fig. 4f), with GV expression confirmed by PCM and TEM (Extended Data Fig. 4g,h). Only 11 colonies (<0.3% after ~35 cell generations) exhibited a mutated phenotype with reduced opacity and GV production (Supplementary Fig. 7a,b). These results indicate that mutational inactivation of GV production is not a major issue for pBAD-bARG_Ser_-AxeTxe EcN under typical conditions.

After establishing construct stability, we characterized the acoustic properties of bARG_Ser_-expressing EcN. For cells induced in liquid culture for 24 hours and suspended at 10^9^ cells per milliliter in agarose phantoms, xAM signal was detected at acoustic pressures above 0.72 MPa, rising with increasing pressure up to the tested maximum of 1.74 MPa (Fig. 2d). The signal remained steady over time at pressures up to 0.96 MPa, above which we observed a slow decrease that followed an exponential decay (Fig. 2e, Supplementary Fig. 8 and Supplementary Note 2), indicating that some of the GVs gradually collapsed despite sustained high xAM signals. We also imaged the cells with parabolic pulses, which can transmit higher pressures than xAM and, thus, can be helpful in vivo to compensate for attenuation at tissue interfaces. When imaged with parabolic B-mode, the GVs started to collapse slowly at 1.02 MPa and more rapidly at 1.33 MPa and above (Fig. 2f). Based on these results, we chose an acoustic pressure of 1.29 MPa for xAM and 1.02 MPa for parabolic AM (pAM) in subsequent experiments to obtain the strongest signals while minimizing GV collapse.

**Extended Data Fig. 5:**
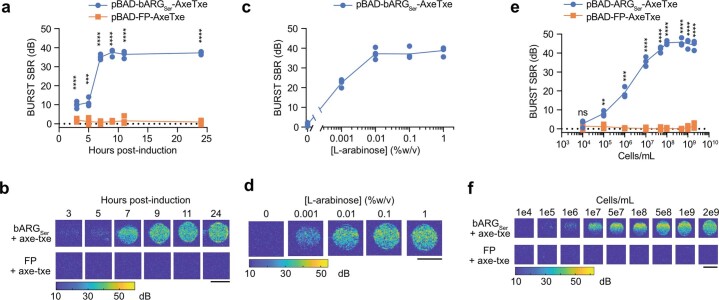
*In vitro* characterizations of bARG_Ser_-expressing EcN with BURST ultrasound imaging. (**a-b**) BURST ultrasound signal-to-background ratio (SBR) versus time after inducing pBAD-bARG_Ser_-AxeTxe and pBAD-FP-AxeTxe EcN strains with 0.1% L-arabinose in liquid culture at 37 °C (p values from left to right: 9.866231E-05, 0.00019939, 5.648535E-09, 9.858233E-09, 1.069657E-07, 1.161991E-09) (a) and the corresponding representative BURST images (b). (**c-d**) BURST ultrasound SBR versus L-arabinose concentration used to induce pBAD-bARG_Ser_-AxeTxe EcN in liquid culture at 37 °C for 24 hours (c) and the corresponding representative BURST images. (**e-f**) BURST ultrasound SBR versus concentration of pBAD-bARG_Ser_-AxeTxe or pBAD-FP-AxeTxe EcN cells induced for 24 hours at 37 °C with 0.1% L-arabinose in liquid culture (p values from left to right: 0.420535, 0.00261501, 0.000335619, 1.899074E-05, 2.348183E-06, 2.055919E-07, 2.793374E-06, 9.045661E-06, 1.487459E-05) (e) and the corresponding representative BURST images (f). Note that the BURST SBR saturated at 7 hours post-induction, 0.01% (w/v) L-arabinose, and 10^8^ cells/mL. All scale bars are 2 mm. For (a-d), cells were normalized to 10^9^ cells/mL in agarose phantoms for ultrasound imaging. For (a, c, e), each point is a biological replicate (n = 4 for a and c; n = 3 for e) that is the average of at least 2 technical replicates and curves indicate the mean. Asterisks represent statistical significance by two-tailed, unpaired Student’s t-tests (**** = p < 0.0001, *** = p < 0.001, ** = p < 0.01, ns = no significance). Source data

Next, to characterize the sensitivity and dynamics of ultrasound contrast in bARG_Ser_-expressing EcN, we measured xAM signal as a function of induction time, inducer concentration and cell concentration. At a density of 10^9^ cells per milliliter, xAM signal could first be observed 7 hours after induction with 0.1% L-arabinose and leveled off by 9 hours after induction (Fig. 2g,h). Keeping the induction time constant at 24 hours while varying the L-arabinose concentration, GV expression was detected with as little as 0.001% L-arabinose, and the highest ultrasound signal was observed for 0.1–1% L-arabinose (Fig. 2i,j). When cells induced for 24 hours with 0.1% L-arabinose were diluted, they were detectable by ultrasound down to 10^7^ cells per milliliter (Fig. 2k,l). Critically, this detection was achieved non-destructively with non-linear imaging compared to previous bacterial ARGs, which required a destructive linear imaging approach^6^. The bARG_Ser_ xAM signal was proportional to the cell concentration between 10^7^ cells per milliliter and 2 × 10^9^ cells per milliliter (Fig. 2k,l). We also imaged the cells using BURST, which provides greater sensitivity at the cost of collapsing the GVs^10^. BURST imaging detected bARG_Ser_ expression as early as 3 hours after induction (Extended Data Fig. 5a,b), with as little as 0.001% L-arabinose (Extended Data Fig. 5c,d) and at a density as low as 10^5^ cells per milliliter (Extended Data Fig. 5e,f).

**Extended Data Fig. 6:**
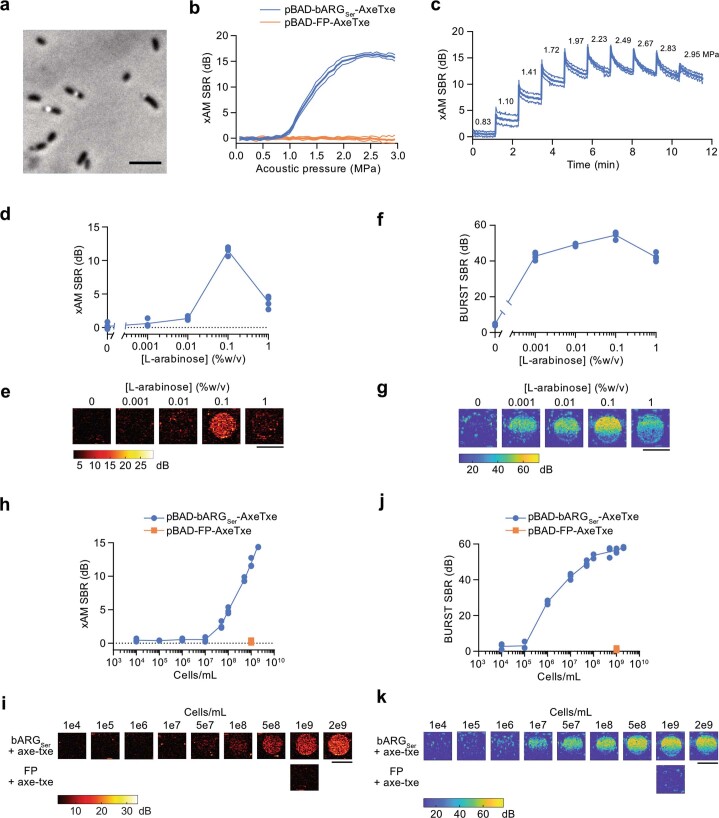
bARG_Ser_ expression and acoustic characterization in *Salmonella enterica* serovar Typhimurium. (**a**) Representative phase contrast microscopy image of bARG_Ser_-expressing *S. Typhimurium* cells (n = 4 biological replicates). (**b**) xAM SBR as a function of transmitted acoustic pressure for bARG_Ser_-expressing and FP-expressing *S. Typhimurium* cells. (**c**) xAM SBRs measured over time when the transmitted acoustic pressure was increased approximately every 70 sec as indicated by the numbers above the curve for bARG_Ser_-expressing *S. Typhimurium*. Ultrasound was applied at a pulse repetition rate of 86.8 Hz. For (a-c), pBAD-bARG_Ser_-AxeTxe *S. Typhimurium* cells were induced with 0.1% (w/v) L-arabinose for 24 hours at 37 °C in liquid culture, and were then diluted to 10^9^ cells/mL in agarose phantoms for ultrasound imaging. Bold lines represent the mean and thin lines represent ± standard deviation; n = 3 biological replicates, each with 2 technical replicates. (**d-g**) xAM ultrasound SBR (d) and corresponding representative ultrasound images (e), and BURST SBR (f) and corresponding representative images (g), using varying L-arabinose concentrations to induce pBAD-bARG_Ser_-AxeTxe *S. Typhimurium* in liquid culture at 37 °C for 24 hours. (**h-k**) xAM ultrasound SBR (h) and corresponding representative ultrasound images (i), and BURST SBR (j) and corresponding representative images (k), of varying concentrations of pBAD-bARG_Ser_-AxeTxe or pBAD-FP-AxeTxe *S. Typhimurium* cells induced for 24 hours at 37 °C with 0.1% (w/v) L-arabinose in liquid culture. Scale bars represent 5 µm in (a) and 2 mm in (e, g, i, k). For (d-g), cells were diluted to 10^9^ cells/mL in agarose phantoms for ultrasound imaging. For (d, f, h, j), each point is a biological replicate (n = 4 for d and f; n = 3 for h and j) that is the average of at least 2 technical replicates, and curves represent the mean. All ultrasound imaging in this figure was performed with an L22-14vX transducer. Source data

Taken together, our in vitro experiments indicated that the reporter gene construct pBAD-bARG_Ser_-AxeTxe is robust and stable in EcN and enables gene expression in these cells to be imaged with high contrast and sensitivity. Similar results were obtained in an attenuated strain of *Salmonella enterica* serovar Typhimurium (Extended Data Fig. 6), another species used in bacterial anti-tumor therapies^49,50^.

### bARG_Ser_ enables in situ imaging of tumor-colonizing bacteria

**Fig. 3:**
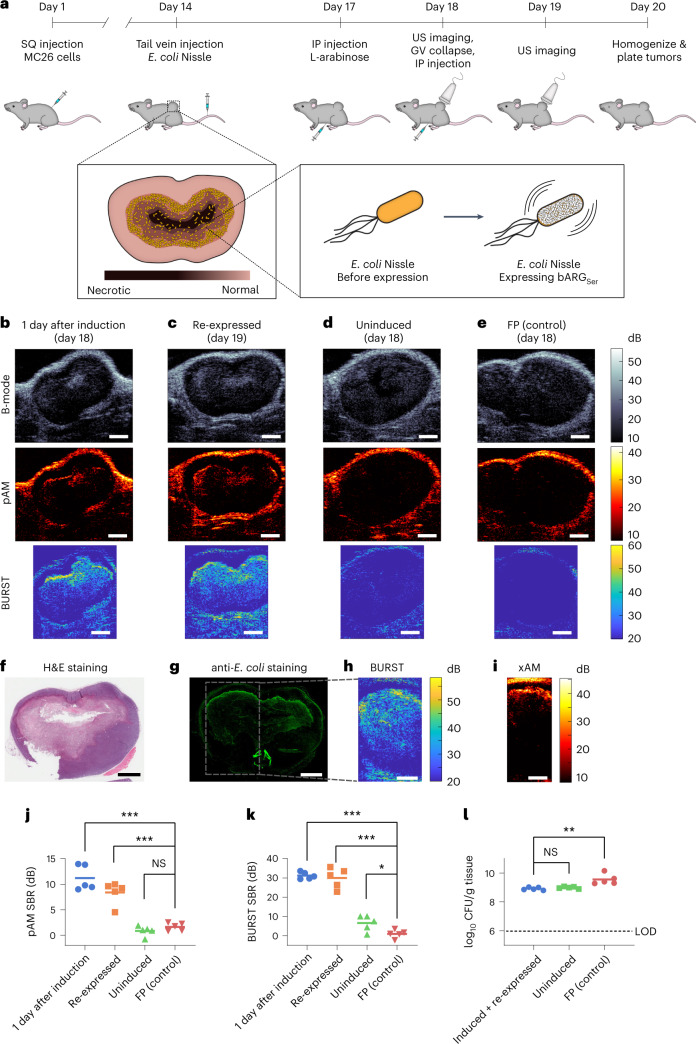
In situ bARG_Ser_ expression enables ultrasound imaging of tumor colonization by EcN. **a**, Diagram of the in vivo protocol for assessing in situ bARG_Ser_ expression in tumors. Mice bearing subcutaneous (SQ) tumors were injected with EcN via the tail vein on day 14. bARG_Ser_ or FP expression was then induced by injecting L-arabinose intraperitoneally (IP) on day 17; the next day, tumors were imaged with ultrasound (US). To check for re-expression, all the GVs in the tumors were collapsed; L-arabinose was re-injected; and tumors were imaged again the next day. On day 20, tumors were homogenized and plated to quantify the levels of EcN colonization. In separate experiments for histology, mice were sacrificed directly after imaging. **b**–**d**, Representative B-mode, pAM and BURST US images of tumors colonized by pBAD-bARG_Ser_-AxeTxe EcN 1 day after induction with L-arabinose (day 18). (**b**), at 1 day after collapse and re-induction (day 19) (**c**) or uninduced on day 18 (**d**). **e**, Representative US images of tumors colonized by pBAD-FP-AxeTxe EcN 1 day after induction with L-arabinose (day 18). **f**,**g**, Optical images of tissue sections stained with H&E (**f**) or anti-*E. coli* antibodies (**g**) from a tumor colonized by pBAD-bARG_Ser_-AxeTxe EcN after imaging on day 19. **h**,**i**, BURST (**h**) and xAM (**i**) images of the same tumor as in **f**,**g**, with the boxed region showing the approximate BURST imaging region in the tissue section. Scale bars in **b**–**i**, 2 mm. **j**,**k**, Quantification of pAM (*P* values: 3.503 × 10^−5^, 0.000209802 and 0.19479281) (**j**) and BURST (*P* values: 8.19948 × 10^−9^, 2.98285 × 10^−6^ and 0.023377359) (**k**) signal-to-background ratios (SBRs) for the conditions in **b**–**e**. **l**, CFUs per gram of tumor tissue on day 20 (*P* values: 0.1911 and 0.0055). For **j**–**l**, points represent each mouse (*n* = 5), and lines represent the mean of each group. The dotted line indicates the limit of detection. Asterisks represent statistical significance by two-tailed, unpaired Student’s *t*-tests (***P < 0.001; **P < 0.01; *P < 0.05; NS, not significant). See Extended Data Fig. 7 for representative xAM ultrasound images for the conditions in **b**–**d** and Supplementary Fig. 9 for more histological images of tissue sections. Source data

**Extended Data Fig. 7:**
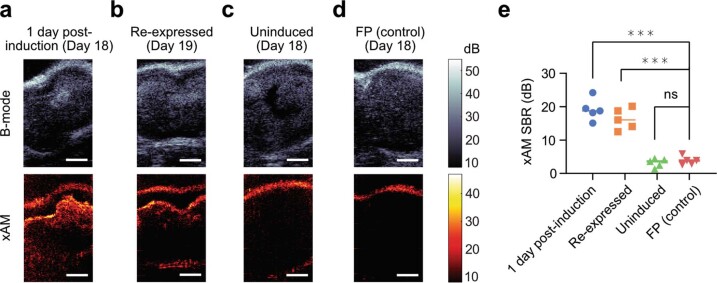
xAM ultrasound imaging of mouse tumors colonized by EcN. (**a-c**) Representative B-mode (top, grayscale) and xAM (bottom, hot-scale) ultrasound images of tumors colonized by pBAD-bARG_Ser_-AxeTxe EcN 1 day after induction with L-arabinose on day 18 (a), 1 day after collapse and re-induction (day 19) (b), or uninduced on day 18 (c). (**d**) Representative ultrasound images of tumors colonized by pBAD-FP-AxeTxe EcN 1 day after induction with L-arabinose on day 18. Scale bars in (a-d) represent 2 mm. (**e**) Quantification of the xAM SBR for the same conditions in (a-d) (p values from left to right: 1.0545E-05, 3.73902E-05, 0.487197948). Each group is n = 5 mice and lines indicate the mean. Asterisks represent statistical significance by two-tailed, unpaired Student’s t-tests (*** = p < 0.001, ns = no significance). See Fig. 3a for the corresponding *in vivo* protocol. Source data

To test the ability of bARG_Ser_ to image the colonization and microscale distribution of bacteria inside tumors, we formed subcutaneous MC26 tumors in mice and intravenously injected EcN cells containing the pBAD-bARG_Ser_-AxeTxe plasmid. Three days after injecting the bacteria, we induced GV expression and imaged the tumors with ultrasound (Fig. 3a). In all tumors colonized by bARG_Ser_-expressing EcN, we observed pAM, BURST and xAM contrast 1 day after induction with L-arabinose (Fig. 3b and Extended Data Fig. 7a). The signals were localized to the core of the tumor and concentrated at the interface between live and necrotic tissue, where the EcN primarily colonized, as confirmed with subsequent tissue histology (Fig. 3f–i and Supplementary Fig. 9). This biodistribution reflects the immune-privileged environment of the necrotic tumor core^45,51^.

After applying 3 MPa of acoustic pressure throughout the tumor to collapse the GVs, we re-injected the mice with L-arabinose and found that similar ultrasound signals could be re-expressed in all tumors colonized by bARG_Ser_-expressing EcN (Fig. 3c and Extended Data Fig. 7b), showing that bARG_Ser_ can be used to visualize dynamic gene expression at multiple timepoints. Absent L-arabinose induction, no xAM or pAM ultrasound signals were observed from bARG_Ser_-containing EcN (Fig. 3d and Extended Data Fig. 7c); likewise, no xAM or pAM ultrasound signals were seen in tumors colonized by FP-expressing EcN (Fig. 3e,j and Extended Data Fig. 7d,e). Low levels of BURST signal were observed in uninduced animals (Fig. 3k), likely due to small amounts of L-arabinose present in the diet combined with the high sensitivity of BURST imaging.

To quantify tumor colonization, on day 20 of the experiment we plated tumor homogenates on selective media. Tumors from all groups of mice contained more than 7 × 10^8^ colony-forming units (CFUs) per gram of tissue (Fig. 3l), indicating that the EcN can persist in tumors at high levels for at least 6 days after intravenous injection regardless of bARG_Ser_ expression, collapse and re-expression. The somewhat higher density of FP-expressing EcN suggests that maintenance of the smaller pBAD-FP-AxeTxe plasmid (7.2 kb versus 23.2 kb for pBAD-bARG_Ser_-AxeTxe), which would impose less burden on the cell^52–55^, may be easier in this in vivo context where oxygen and other nutrients are limited. Negligible mutational silencing was observed in EcN plated from tumor samples (Supplementary Fig. 10a–c).

Taken together, our in vivo experiments with EcN demonstrate that bARG_Ser_ expression enables stable, non-destructive acoustic visualization of the microscale distribution of these probiotic agents in a therapeutically relevant context.

### GV gene cluster with non-linear contrast in mammalian cells

**Fig. 4:**
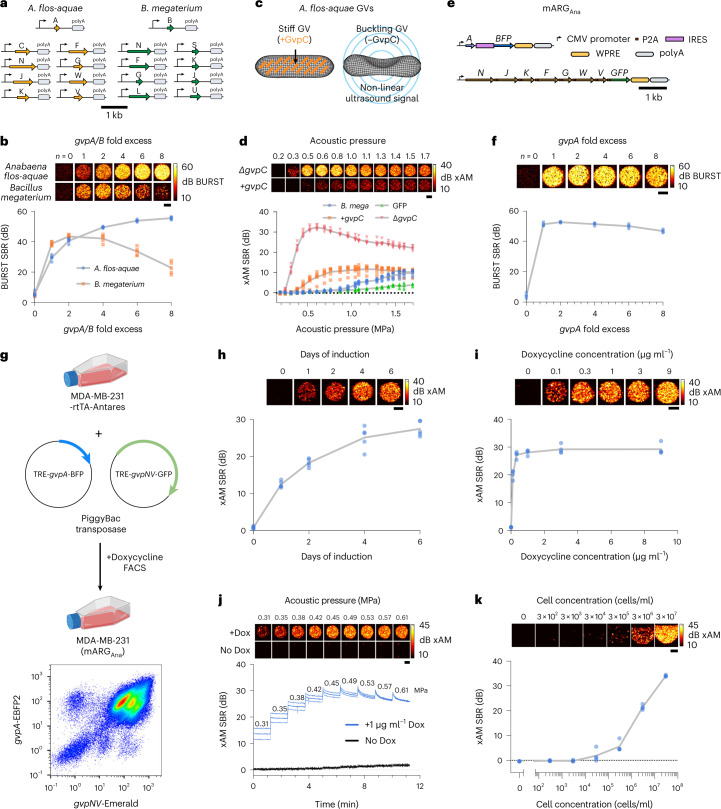
Heterologous expression of the *A. flos-aquae* GV gene cluster in mammalian cells. **a**, Schematic of the codon-optimized monocistronic plasmid sets used in this study. **b**, Representative BURST images (top) and signal-to-background ratio (SBR) quantification (*n* = 5, bottom) of transient GV expression in HEK293T cells 3 days after co-transfection of mixtures with varying *gvpA/B* fold excess relative to their respective assembly factor plasmids. **c**, Diagram of GV structure with GvpC highlighted in orange. **d**, Representative xAM ultrasound images (top) and SBR quantification (*n* = 6, bottom) of transient co-transfection experiments of *A. flos-aquae* GV plasmids (four-fold *gvpA* excess) with and without *gvpC* at varying acoustic pressures. *B. megaterium* GV (at two-fold *gvpB* excess) and GFP expression is included for quantitative comparison. **e**, Schematic of the mARG_Ana_ polycistronic plasmids. **f**, Representative BURST images (top) and SBR quantification (*n* = 4, bottom) of transient GV expression in HEK293T cells 3 days after co-transfection of mARG_Ana_ mixtures with varying *gvpA* fold excess relative to the assembly factor plasmid. **g**, Schematic of MDA-MB-231-mARG_Ana_ engineering (created with BioRender and FlowJo). The final population was ~95% double-positive for *gvpA* and *gvpNJKFGWV* expression. **h**, Representative xAM images (top) and SBR quantification (*n* = 5, bottom) of MDA-MB-231-mARG_Ana_ cells imaged at 0.54 MPa after 1, 2, 4 and 6 days of 1 µg ml^−1^ doxycycline induction. **i**, Representative xAM images (top) and SBR quantification (*n* = 4, bottom) of MDA-MB-231-mARG_Ana_ cells imaged at 0.42 MPa as a function of doxycycline concentration after 4 days of expression. **j**, Representative xAM images (top) and SBR quantification (*n* = 4, bottom) of induced and uninduced MDA-MB-231-mARG_Ana_ cells as a function of time while imaged with sequentially increased acoustic pressures, starting at 0.31 MPa. **k**, Representative xAM images (top) and SBR quantification (*n* = 4, bottom) of induced MDA-MB-231-mARG_Ana_ cells at 0.61 MPa as a function of cell concentration. Limit of detection was 300,000 cells per milliliter (*P* = 0.0181) by unpaired one-sided *t*-test. For **j**, thick lines represent the mean of four replicates, and thin lines represent ± standard deviation. For **b**,**d**,**f**,**h**,**i**,**k**, gray lines connect the means of the replicates. All ultrasound image scale bars represent 1 mm. Dox, doxycycline; FACS, fluorescence-activated cell sorting. Source data

Next, we developed improved ARGs for mammalian cells. The first-generation mammalian ARGs were based on the GV gene cluster from *B. megaterium* (referred to here as mARG_Mega_). mARG_Mega_ expression could be detected only with destructive collapse-based imaging due to a low level of GV expression and the lack of non-linear contrast^7^. Moreover, successful use of mARG_Mega_ as a reporter gene required monoclonal selection of transduced cells and their treatment with a broadly acting histone deacetylase inhibitor^7^. Seeking mARGs that are expressed more robustly and produce non-linear contrast, we cloned mammalian versions of the genes contained in each of the three clusters that produced non-linear signal in *E. coli* at 37 °C: *Serratia*, *A. flos-aquae* and *A. flos-aquae/B. megaterium* (Fig. 1d,e). Equimolar transient co-transfections of the monocistronic genes derived from each gene cluster into HEK293T cells yielded detectable BURST signal only for the *A. flos-aquae* gene cluster and mARG_Mega_^7^ (Fig. 4a,b; ‘one-fold excess’).

**Extended Data Fig. 8:**
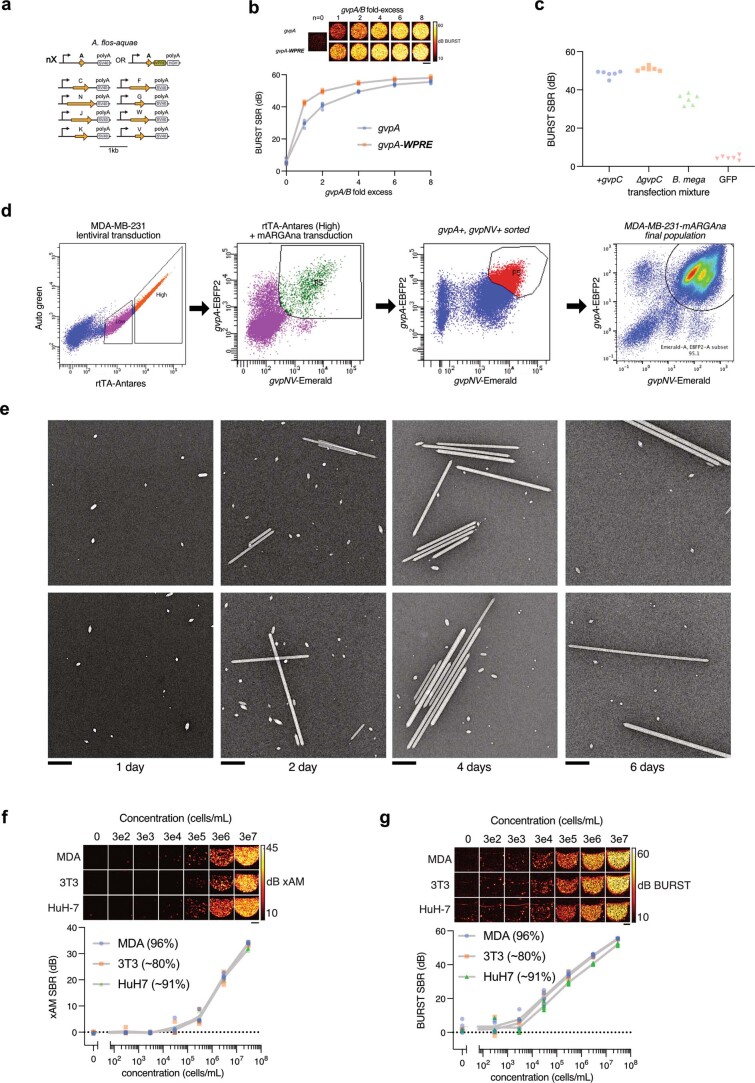
Additional data on heterologous expression of the *A. flos-aquae* GV gene cluster in mammalian cells. (**a**) Schematic of the codon-optimized *A. flos-aquae* monocistronic plasmid set used in this study. (**b**) Representative BURST images (top) and SBR quantification (n = 5, bottom) of transient GV expression in HEK293T cells 3 days after co-transfection of mixtures with varying *gvpA* fold excess relative to their respective assembly factor plasmids, with and without WPRE elements on the *gvpA* DNA. Gray lines connect the means of replicates. (**c**) BURST SBR quantification (n = 6) of HEK293T cells expressing constructs tested in Fig. 4d. (**d**) FACS and flow cytometry data for production of the MDA-MB-231-mARG_Ana_ cell line. rtTA expressing ‘High’ cells were collected for subsequent mARG/transposase transduction. Cells expressing *gvpA* and *gvpNV* were sorted twice to arrive at the final ~95% pure population. (**e**) TEM images of GVs purified from MDA-MB-231-mARG_Ana_ detergent lysates after 1, 2, 4 and 6 days of 1 µg/ml doxycycline induction (n = 35 micrographs for day 1, 29 for day 2, 32 for day 4 and 7 for day 6). Scalebars represent 0.5 µm. (**f**) Representative xAM images (top) and SBR quantification (n = 4 biologically independent samples examined in one experiment, bottom) of induced MDA-MB-231-mARG_Ana_, 3T3-mARG_Ana_ and HuH7-mARG_Ana_ cells at 0.61 MPa as a function of cell concentration. Limit of detection was 300k cells/mL for MDA-MB-231 (p = 0.0181) and 3T3 (p = 0.0321), and 30k cells/mL for HuH7 (p = 0.0158) by unpaired one-sided t-tests. (**g**) Representative BURST images (top) and SBR quantification (n = 4, bottom) of induced MDA-MB-231-mARG_Ana_, 3T3-mARG_Ana_ and HuH7-mARG_Ana_ cells as a function of cell concentration. Limit of detection was 30k cells/mL for MDA-MB-231 (p = 0.011) and HuH7 (p = 0.006), and 3k cells/mL for 3T3 (p = 0.027) by unpaired one-sided t-tests. For (f) and (g), gray lines connect the means of the replicates and scalebars represent 1 mm. Percentages in parentheses represent mARG_Ana_-positive cells determined by flow cytometry. Source data

Given the multiple *gvpA* copies contained in the native *A. flos-aquae* GV operon^8^, we hypothesized that expressing GvpA at a higher stoichiometry relative to the other genes in this cluster could improve GV expression. To test this possibility, we titrated the amount of *gvpA* plasmid in the *A. flos-aquae* plasmid set while keeping the DNA amount corresponding to other genes constant (the total DNA level was kept constant with a padding vector). We found that the BURST signal increased monotonically with increasing *gvpA* up to eight-fold *gvpA* excess (Fig. 4b). In contrast, the signal peaked at two-fold excess of the homologous protein *gvpB* for the *B. megaterium* cluster. This suggests that the assembly factors in *the A. flos-aquae* gene cluster may be more efficient at using GvpA to form GVs or that excess GvpA may pose less of a burden to cells than excess GvpB. To further improve GvpA expression, we stabilized the *gvpA* transcript with WPRE-hGH polyA elements^56^, which resulted in peak signal at lower *gvpA*:chaperone ratios (Extended Data Fig. 8a,b).

We next looked for non-linear ultrasound contrast. GvpC is a minor structural protein in *A. flos-aquae* GVs that mechanically reinforces the GV shell (Fig. 4c)^8^. Chemical removal of GvpC from purified GVs in vitro enhances non-linear ultrasound scattering by allowing the GVs to deform more strongly in response to acoustic pressure^33,57,58^. When we omitted *gvpC* from our mammalian co-transfection mixture, we observed a marked enhancement of non-linear signal in xAM, peaking at a transmit pressure of ~0.6 MPa (Fig. 4d). By comparison, transfections including *gvpC* produced a much weaker xAM signal, and *B. megaterium* plasmids and GFP-expressing cells did not produce appreciable non-linear contrast at any pressure. At 0.6 MPa, the *A. flos-aquae* gene cluster without GvpC had a 38.8-fold stronger xAM signal compared to the *B. megaterium* construct. The omission of *gvpC* did not substantially alter BURST contrast (Extended Data Fig. 8c).

To create a convenient vector for mammalian expression of *A. flos-aquae* GVs, we constructed a polycistronic plasmid linking the assembly factor genes *gvpNJKFGWV* through P2A co-translational cleavage elements. *gvpA* was supplied on a separate plasmid to enable stoichiometric tuning. The *gvpA* and *gvpNJKFGWV* plasmids were labeled with IRES-BFP and P2A-GFP, respectively, to allow for fluorescent analysis and sorting. Both transcripts were driven by cytomegalovirus (CMV) promoters. We termed this pair of plasmids mARG_Ana_: mammalian ARGs adapted from *A. flos-aquae* (Fig. 4e). mARG_Ana_ produced BURST ultrasound contrast in HEK293T cells transiently co-transfected with a one-fold to six-fold molar excess of *gvpA* (Fig. 4f).

To produce a stable cancer cell line expressing mARG_Ana_, we cloned our polycistronic constructs into PiggyBac integration plasmids under a doxycycline-inducible TRE promoter^59,60^. As a clinically relevant cancer model, we chose the human breast cancer cell line MDA-MB-231, which is widely used in tumor xenograft studies. We engineered these cells to constitutively co-express the rtTA transactivator and Antares optical reporter^61^, transduced them with a mixture of mARG_Ana_ and PiggyBac transposase plasmids at a 2:1 (*gvpA:gvpNJKFGWV*) molar ratio and fluorescently sorted for co-expression of Antares, GFP and BFP (Fig. 4g and Extended Data Fig. 8d).

The resulting polyclonal MDA-MB-231-mARG_Ana_ cells showed xAM contrast after a single day of doxycycline induction, which increased through day 6 (Fig. 4h). We confirmed the expression of GVs by electron microscopy (Extended Data Fig. 8e). The ultrasound signal increased steeply with increasing doxycycline doses up to 1 µg ml^−1^ and then saturated (Fig. 4i). xAM signal was detected starting from an acoustic pressure of 0.31 MPa, whereas uninduced control cells did not produce signal at any pressure (Fig. 4j). At pressures above 0.42 kPa, the xAM signal gradually decreased over time, indicating the partial collapse of GVs. We chose 0.42 MPa as the xAM imaging pressure for subsequent experiments, providing the optimal balance of signal stability and strength.

We obtained similar results with mouse 3T3 fibroblasts and human HuH7 hepatocytes, with both cell lines detectable with similar sensitivity to MDA-MB-231 cells when expressing mARG_Ana_ (Fig. 4k and Extended Data Fig. 8f). At 300,000 cells per milliliter for MDA-MB-231 and 3T3 and at 30,000 cells per milliliter for HuH7, the non-destructive xAM detection limits of these cells surpassed the destructive imaging sensitivity of first-generation mARG_Mega_ by 1–2 orders of magnitude^7^. With BURST imaging, mARG_Ana_ cells could be detected still more sensitively, at concentrations down to 3,000 cells per milliliter for 3T3 cells and 30,000 cells per milliliter for MDA-MB-231 and HuH7 cells (Extended Data Fig. 8g).

### mARG_Ana_-based imaging of in vivo gene expression patterns

**Fig. 5:**
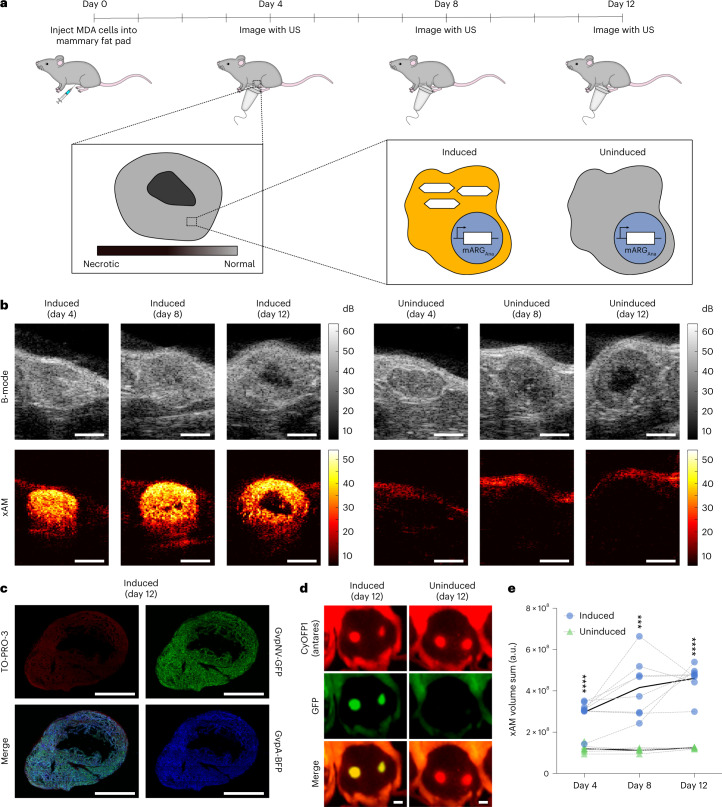
In situ mARG_Ana_ expression enables non-destructive ultrasound imaging of orthotopic tumors. **a**, Diagram of the in vivo protocol for assessing in situ mARG_Ana_ expression in orthotopic tumors. Mice were injected bilaterally in the 4th mammary fat pads with engineered MDA-MB-231-mARG_Ana_ human breast adenocarcinoma cells on day 0. mARG_Ana_ expression was induced by regular intraperitoneal doxycycline injections starting from the day of tumor injections. Tumors were imaged with ultrasound (US) after 4, 8 and 12 days of expression. **b**, Representative middle sections of B-mode and xAM US tomograms of MDA-MB-231 mARG_Ana_ tumors induced with doxycycline (left; *n* = 8 tumors from four mice) and uninduced control (right; *n* = 7 tumors from four mice on day 4 and *n* = 5 tumors from four mice on days 8 and 12) imaged on days 4, 8 and 12. Scale bars, 2 mm. See Supplementary Video 1 for the full US tomogram of the induced tumor at day 12. **c**, Fluorescence micrograph of a 100-μm-thin tumor section (representative of *n* = 2 tumors). Green color shows GFP fluorescence; blue color shows BFP fluorescence; and red color shows TO-PRO-3 nuclear stain. See Extended Data Fig. 9 for the uninduced control (*n* = 1 tumor). Scale bars, 2 mm. **d**, Whole-animal fluorescence imaging of induced (left; *n* = 8 tumors from four mice) and uninduced (right; *n* = 5 tumors from three mice) tumors after 12 days of expression. All tumors are constitutively expressing CyOFP1 (Antares, red), whereas mARG_Ana_ expression is linked to expression of GFP (green). The left (reader’s right) tumors are shown in **b**. Scale bars, 5 mm. **e**, 3D sum of xAM signal from US tomograms of induced (*n* = 8) and uninduced (*n* = 7 tumors from four mice on day 4 and *n* = 5 tumors from four mice on days 8 and 12) tumors from one experiment, plotted on a linear scale in arbitrary units (a.u.). Black curves connect the means, and gray curves connect points for each mouse. Asterisks represent statistical significance by two-tailed, unpaired Student’s *t*-tests between induced and uninduced conditions (*P* values from left to right: 0.000622, 0.001554 and 0.001554) (****P < 0.0001 and ***P < 0.001). Source data

**Extended Data Fig. 9:**
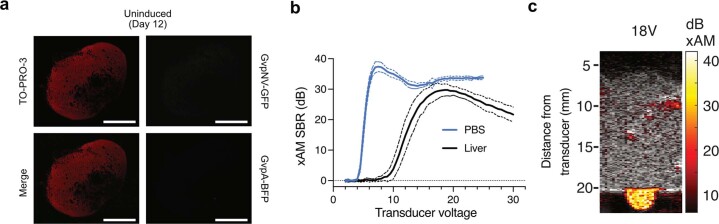
Tumor histology and imaging through thick tissue. (**a**) Fluorescence micrograph of a 100 μm-thick tissue section from an uninduced tumor. The red color shows TO-PRO-3 nuclear stain; GFP and BFP fluorescence were not observed for this control tumor. Scale bars represent 2 mm. (**b**) Quantification of xAM SBR of MDA-MB-231-mARG_Ana_ cells imaged under >1 cm of beef liver tissue (n = 7) or under PBS (n = 4) as a function of transducer voltage. Thick lines represent the mean of replicates and thin lines represent ± standard deviation. (**c**) Representative xAM/B-mode overlay of MDA-MB-231-mARG_Ana_ cells imaged under liver tissue using L10-5v transducer at 18 V for xAM. Source data

We next tested the ability of mARG_Ana_ to reveal the spatial distribution of gene expression in tumor xenografts. We formed orthotopic tumors by injecting MDA-MB-231-mARG_Ana_ cells bilaterally in the 4th mammary fat pads of female immunocompromised mice. The mice were then split into doxycycline-induced and uninduced groups. We acquired ultrasound images of the tumors as they grew, with three imaging sessions distributed over 9 days (Fig. 5a). All induced tumors produced bright and specific xAM contrast starting from the first timepoint (day 4), whereas the uninduced tumors did not (Fig. 5b). The acquisition of adjacent planes allowed three-dimensional (3D) visualization of expression patterns (Supplementary Video 1). The non-linear xAM signal was highly specific to the viable tumor cells, being absent outside tumor boundaries and within the necrotic cores of the larger tumors. This observed spatial pattern was corroborated by fluorescence microscopy of fixed tumor sections obtained on day 12 (Fig. 5c and Extended Data Fig. 9a), confirming the ability of mARG_Ana_ to report microscale patterns of gene expression non-invasively in living animals. In contrast, in vivo fluorescence images lacked information about the spatial distribution of gene expression within the tumor (Fig. 5d). The induced tumors had significantly higher total signal than the uninduced controls at all timepoints (Fig. 5e).

To demonstrate mARG_Ana_ imaging at depths beyond what can be easily shown in mice, we successfully imaged MDA-MB-231-mARG_Ana_ cells through a slab of beef liver thicker than 1 cm (Extended Data Fig. 9b,c).

### mARG_Ana_ imaging enables real-time ultrasound-guided biopsy

**Fig. 6:**
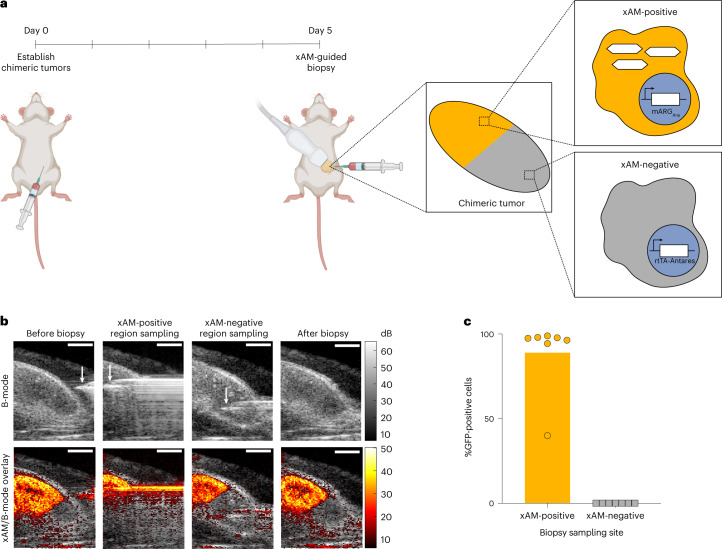
xAM imaging of mARG_Ana_ enables ultrasound-guided genetically selective tumor biopsy. **a**, Diagram of the in vivo protocol for establishing chimeric tumors, in situ expression of GVs and tumor biopsy. Chimeric tumors were established on day 0. mARG_Ana_ expression was induced by regular intraperitoneal doxycycline injections starting from the day of tumor injections. Tumors were imaged with ultrasound and biopsied after 5 days of expression. **b**, Representative B-mode and xAM images of an ultrasound-guided biopsy procedure. Scale bars, 2 mm. See Supplementary Videos 2–5 for the full ultrasound tomogram of the induced chimeric tumor, 3D reconstruction of the chimeric tumor as well as the videos of the full procedure. **c**, Results of flow cytometric analysis of biopsied samples from xAM-positive and xAM-negative regions of chimeric tumors (*n* = 7 tumors from four mice). See Supplementary Fig. 11 for the flow cytometric gating strategy. Bar height represents the mean, and circles represent individual data points. Source data

One of the most common uses of ultrasound is to spatially guide procedures such as biopsies. ARGs provide the possibility for such procedures to be targeted based on gene expression. However, because real-time procedure guidance requires non-destructive imaging, such guidance was not possible with previous ARGs. To demonstrate an ultrasound-guided procedure with these ARGs, we generated chimeric tumors in mice via adjacent subcutaneous injection of mARG_Ana_-expressing and Antares-expressing MDA-MB-231 cells (Fig. 6a). After 3D tomograms confirmed chimeric tumor composition (Supplementary Videos 2 and 3), we targeted fine needle aspiration biopsies to either the xAM-positive or the xAM-negative regions of each tumor (Fig. 6b and Supplementary Videos 4 and 5). Flow cytometry showed a high success rate in obtaining cells with the expected mARG_Ana_ positivity or negativity (Fig. 6c and Supplementary Fig. 11).

## Discussion

Our results establish two ARG constructs—bARG_Ser_ and mARG_Ana_—that provide unprecedented real-time detection sensitivity and specificity when expressed in bacteria and mammalian cells. These gene clusters produce bright non-linear ultrasound contrast when expressed in situ, either by bacterial agents colonizing the necrotic core of a tumor or by the tumor cells themselves. Their contrast enables the dynamic monitoring of the precise locations and transcriptional activities of these cells over multiple days. Furthermore, real-time non-destructive imaging of ARGs enables ultrasound-guided procedures, such as biopsies, targeted to genetically defined cells.

With the improvements described here, we anticipate that our ARGs will be useful for various applications that demand the non-invasive imaging of cells several millimeters or deeper inside the body. bARGs could be used to track therapeutic bacteria as they home to and proliferate in tumors or other target organs to assess whether the therapy is working as designed^62–65^. Furthermore, knowing their spatial distribution can help guide tumor-focused interventions, such as focused ultrasound^66–68^. Other potential applications include microbiome research and tracking of probiotics designed to diagnose or treat GI conditions^69,70^.

In addition, mARGs could be used to visualize the growth and viability of tissues, such as tumors, and precisely sample their contents under ultrasound guidance. Similar approaches could be applied to the immune system, brain and organismal development. Moreover, mARG expression could enable the tracking and sampling of therapeutic cells, such as T cells or stem cells. The fact that the mARGs are based on *A. flos-aquae* GVs will allow these ARGs to benefit from extensive molecular engineering of these GVs^2,6,57,71^ and development of acoustic biosensors of cellular signals such as enzyme activity^71^.

The envisioned applications of both bacterial and mammalian ARGs will benefit from the relative simplicity and low cost of ultrasound compared to other non-invasive techniques, such as nuclear imaging and magnetic resonance imaging (MRI), while providing in vivo resolution and potential for human translatability beyond what is currently possible with optical methods. In making cells detectable with ultrasound imaging, ARGs help pure ultrasound rival photoacoustic tomography, which has a larger number of contrast agents but is technically more complex to implement^72^.

Additional improvements to the technology can be envisaged. First, although expression kinetics on the order of 1 day, as observed with ARGs, are acceptable in numerous scenarios, such as tumor growth, mammalian development and stem cell expansion and migration, they are slower than those of fluorescent proteins^73,74^. Faster expression could potentially be achieved by pre-expressing some of the genes in the ARG cluster (for example, assembly factors) and conditionally expressing the remainder (for example, structural proteins). Second, the ability to multiplex different ‘colors’ of ARGs non-destructively would enable discrimination between different mammalian or bacterial cell types in a tissue. This could be accomplished by engineering versions of GvpC that respond differently to pressure^57^. Third, it would be helpful to reduce the size of the *Serratia* GV gene cluster to make it easier to clone and incorporate with other genetic elements. Similarly, consolidating the two-part mARG_Ana_ construct into a single plasmid would increase transfection efficiency, and shortening it could facilitate viral packaging for delivery to endogenous cells. Fourth, although the in vivo expression of both bARG_Ser_ and mARG_Ana_ yielded strong and specific ultrasound signals without obvious effects on cell migration and growth, strategies to reduce construct size and tightly regulate ARG expression could be beneficial to reducing burden under more stringent or resource-limited conditions. Finally, although mice are a key model in biomedical research and an established proving ground for reporter gene technologies^61,75–78^, future work is needed to test ARGs in additional species. The ability of improved ARGs to function in cells from two different mammalian species and two different bacterial species is an encouraging sign for their broader utility.

Out of practical necessity, our phylogenetic screen subsampled the available genetic space, and testing additional GV-encoding gene clusters could reveal ARGs with new or further-improved properties. Improvements can also be made in the screening strategy by synthesizing multiple versions of each putative gene cluster to eliminate potential regulatory genes^9,34,36–38^ and screening a larger number of clusters in higher throughput. It is likely that GV gene clusters from certain species will be more compatible with specific heterologous hosts due to similarity in growth temperature, turgor pressure and host factors, making it useful to perform genetic screens with new species of interest for imaging.

Just as improvements to and adaptations of fluorescent proteins enabled a wide range of microscopy applications that were mere speculations when GFP was first harnessed as a biotechnology, the systematic development of ARGs could help realize the promise of sensitive, high-resolution non-invasive imaging of cellular function inside intact mammals.

## Methods

### Genomic mining of ARG clusters

A literature search was conducted to find papers reporting the production of GVs in any species. Search terms included ‘gas vesicle,’ ‘gas vacuole,’ ‘aerotope’ and ‘aerotype.’ All species found are listed in Supplementary Table 1. If the report did not include a strain name, then any available 16S rRNA gene sequence was used (as it was assumed that any other strain of the same species would fall in the same place on the phylogenetic tree), but no GV gene cluster sequence was used (even if it was available for one or more strains of that species), because it was found during our analysis that (1) several reports describe species for which some strains produce GVs but others do not and (2) comparison of GV gene cluster sequences of multiple strains of the same species almost always showed differences—often very significant ones. Furthermore, even if a reference stating that a given organism produced GVs was not available, 16S rRNA gene sequences from all members of the following genera were included, because GV production is a diacritical taxonomic feature for these genera: *Dolichospermum*^79^, *Limnoraphis*^80^ and *Planktothrix*^81^.

GV clusters were identified in genomes through a combination of annotations and sequence similarity to known *gvp* genes. However, there were two challenges in identifying all *gvp*s in a given genome: (1) there is little to no annotation for many *gvp*s and (2) GV gene clusters are not always contiguous in genomes, and *gvp*s can occasionally be found hundreds of kilobases away from the main cluster(s). We attempted to select only ‘well-behaved’ GV clusters for testing (that is, ones in which all *gvp*s identified in that genome were present in clusters, and these clusters contained a minimum of non-*gvp* genes, which could increase the metabolic burden of cluster expression without increasing GV yield), but it is possible that, even for these clusters, some *gvp*s were not cloned.

Of our list of 288 strains reported to form GVs, 270 had 16S rRNA gene sequences available (Supplementary Table 1). These were downloaded from the National Center for Biotechnology Information (NCBI) (https://www.ncbi.nlm.nih.gov/sites/batchentrez) using a custom Python script, and a multiple sequence alignment was constructed using Clustal Omega^82^. This alignment was used to generate a phylogenetic tree file using ClustalW2 (ref. ^83^), which was rendered using Evolview^84^. Only unique species are displayed in the phylogenetic trees in Fig. 1a and Supplementary Fig. 1.

### Bacterial plasmid construction and molecular biology

Organisms were obtained from commercial distributors as indicated in Supplementary Table 2. If an organism was shipped as a liquid culture, the culture was centrifuged and the pellet resuspended in ddH_2_O, as it was found that even trace amounts of certain culture media could completely inhibit polymerase chain reaction (PCR). Fragments were amplified by PCR using Q5 polymerase and assembled into a pET28a(+) vector (Novagen) via Gibson assembly using reagents from New England Biolabs (NEB). Subcloning and other modifications to plasmids were also performed with Gibson assembly using reagents from NEB. Assemblies were transformed into NEB Stable *E. coli*. All constructs were verified by Sanger sequencing.

*Halobacterium salinarum* has two chromosomal GV gene clusters (plus additional plasmid-borne ones), which were cloned and tested separately. *Methanosarcina vacuolata* has only one cluster, but, whereas its genome sequence in the NCBI database has two copies of *gvpA1* and one copy of *gvpA2*, our genomic PCR yielded a product with only one copy of *gvpA1*. In a second cloning step, we added a copy of *gvpA2* to the cloned cluster. Although we were able to PCR *gvpA2* from the genome, it was not contiguous with the rest of the cluster. Therefore, we speculate that either there was an error in the assembly of the genome sequence (likely caused by the high sequence similarity of the *gvpA* genes) or that the genotype of our strain differs slightly from that of the strain sequenced.

### In vitro bacterial expression of ARGs

For initial testing, all constructs were expressed in BL21(DE3) *E. coli* (NEB). Fifty microliters of electrocompetent *E. coli* were transformed with 1.5 μl of purified plasmid DNA (EconoSpin 96-well filter plate, Epoch Life Science), and 1 ml of SOC medium (NEB) was added immediately after electroporation. These cultures were incubated at 37 °C for 2 hours, and 150 μl was inoculated into larger 1.5-ml LB cultures containing 100 µg ml^−1^ of kanamycin and 1% (w/v) glucose (for catabolite repression^85^ of the BL21(DE3) PlacUV5 promoter) in a deep-well 96-well plate and grown overnight in a shaking incubator at 30 °C. Square dual-layer LB agar plates were prepared with varying concentrations of IPTG and 100 µg ml^−1^ of kanamycin in the bottom layer and 1% (w/v) glucose and 100 µg ml^−1^ of kanamycin in the top layer. Each layer was 25 ml in volume, and the top layer was poured within 30 minutes of plating. LB agar was incubated at 60 °C for 12–36 hours after dissolution to allow it to de-gas. After the agar solidified, plates were dried at 37 °C to remove all condensation on the top layer that would cause the bacterial patches to run together. A multichannel pipette was used to thoroughly mix overnight cultures and drop 1 μl of each culture onto the surface of the dual-layer plates, with care taken to avoid puncturing the agar that results in artifacts during ultrasound scans. Low-retention pipette tips were used, as it was found that the small volumes of culture would wet the outsides of standard pipette tips, resulting in inaccurate volume dispensing. Patches were allowed to dry completely before plates were flipped and incubated at 37 °C for 24 hours or 30 °C for 48 hours.

For in vitro expression experiments in EcN, the appropriate plasmids were first transformed via electroporation, and the outgrowth was plated on LB (Miller) agar plates with the appropriate antibiotic (25 μg ml^−1^ of chloramphenicol or 50 μg ml^−1^ of kanamycin) and 1% (w/v) glucose. The resulting colonies were used to inoculate 2 ml of LB (Miller) medium with the appropriate antibiotic and 1% (w/v) glucose, and these cultures were incubated at 250 r.p.m. and 37 °C overnight. Glycerol stocks were prepared by mixing the overnight cultures in a 1:1 volume ratio with 50% (v/v) glycerol and storing at −80 °C. The night before expression experiments, glycerol stocks were used to inoculate overnight cultures (2 ml of LB medium with the appropriate antibiotic and 1% (w/v) glucose), which were incubated at 37 °C and shaken at 250 r.p.m. For expression on solid media, 1 μl of overnight culture was dropped onto square dual-layer LB agar plates with 2× the final inducer (IPTG, aTc or L-arabinose) concentration in the bottom layer, 1% (w/v) glucose in the top layer and the appropriate antibiotic in both layers (50 μg ml^−1^ of chloramphenicol or 100 μg ml^−1^ of kanamycin). Plates were allowed to dry and then inverted and incubated at 37 °C for 24 hours before imaging with ultrasound. For expression in liquid media, 500 μl of each overnight culture was used to inoculate 50 ml of LB supplemented with 0.4% (w/v) glucose and 25 μg ml^−1^ of chloramphenicol in 250-ml baffled flasks. Cultures were incubated at 37 °C and 250 r.p.m. until reaching an OD_600_ of 0.1–0.3. At this point, cultures were induced by addition of L-arabinose and placed back at 37 °C and 250 r.p.m. For time titration experiments, 0.1% (w/v) L-arabinose was used for induction, and 0.5 ml of each culture was removed at 0, 1, 3, 5, 7, 9, 11 and 24 hours after induction for OD_600_ and ultrasound measurements. For L-arabinose titration experiments, L-arabinose concentrations ranging from 0 to 1% (w/v) were used for induction, and cultures were incubated for 24 hours at 37 °C and 250 r.p.m. after addition of L-arabinose before ultrasound imaging. For cell concentration titration experiments, cultures were incubated for 24 hours at 37 °C and 250 r.p.m. after addition of 0.1% (w/v) L-arabinose before ultrasound imaging. All cultures were stored at 4 °C or on ice until casting in phantoms and imaging with ultrasound. In all liquid culture experiments, cultures were prescreened for the presence of GVs by phase-contrast microscopy before being imaged with ultrasound.

In vitro expression experiments in *S. Typhimurium* were performed as described above for EcN, except plasmids were transformed into an attenuated version of *Salmonella enterica* serovar Typhimurium strain SL1344 (ref. ^49^); 2×YT medium was used instead of LB medium; and induction in liquid culture was performed by adding L-arabinose to the medium at the time of inoculation at an OD_600_ of 0.05. Ultrasound images of bacteria expressing ARGs on solid media and in liquid culture were acquired as described in Supplementary Note 1.

To assess plasmid stability of pBAD-bARG_Ser_-AxeTxe in EcN, the glycerol stock of this strain was used to inoculate 2 ml of LB (Miller) supplemented with 25 μg ml^−1^ of chloramphenicol and 1% (w/v) glucose, and this culture was incubated at 37 °C and 250 r.p.m. overnight. Then, 20 μl of the overnight culture was subcultured into 2 ml of LB with 25 μg ml^−1^ of chloramphenicol, 2 ml of LB without antibiotics and 2 ml of LB without antibiotics and with 0.1% (w/v) L-arabinose, each in quadruplicate. Every 24 hours, 20 μl of each culture was subcultured into fresh media of the same conditions. All cultures were incubated at 37 °C and 250 r.p.m. On days 1–3, 5 and 7, serial dilutions of each culture were plated on LB agar without antibiotics, LB agar with 25 μg ml^−1^ of chloramphenicol and LB agar with 25 μg ml^−1^ of chloramphenicol + 0.1% (w/v) L-arabinose + 0.4% (w/v) glucose. Plates were incubated at 37 °C for at least 16 hours, and colonies were counted and screened manually. Plasmid retention was assessed by taking the ratio of CFUs on LB agar plates with chloramphenicol to CFUs on LB agar plates without antibiotics. The presence of mutations that disrupt the ability to express functional bARG_Ser_ was assessed by a loss of colony opacity on LB agar plates with 25 μg ml^−1^ of chloramphenicol + 0.1% (w/v) L-arabinose + 0.4% (w/v) glucose.

### Microscopy of bacteria

For TEM imaging, cells expressing GVs were diluted to OD_600_ ~1 in 10 mM HEPES (pH 7.5) or culture media. Then, 3 µl of the sample was applied to a freshly glow-discharged (Pelco EasiGlow, 15 mA, 1 minute) formvar/carbon-coated, 200 mesh copper grid (Ted Pella) for 1 minute before being reduced to a thin film by blotting. Grids with cells were washed three times in 10 mM HEPES (pH 7.5), blotted, air-dried and imaged without stain. Image acquisition was performed using a Tecnai T12 (FEI, now Thermo Fisher Scientific) electron microscope operated at 120 kV, equipped with a Gatan Ultrascan 2,000 × 2,000 CCD camera.

For PCM imaging, cells expressing GVs were scraped off from plates and re-suspended in PBS at an OD_600_ of 1–2, or liquid cultures were used directly. Suspensions were transferred to glass slides, and PCM images were acquired using a Zeiss Axiocam microscope with a ×40 Ph2 objective.

### In vivo bacterial ARG expression

All in vivo experiments were performed under protocol 1735 or 1761, approved by the Institutional Animal Care and Use of Committee of the California Institute of Technology. Animals were housed in a facility maintained at 71–75 °F and 30–70% humidity, with a lighting cycle of 13 hours on and 11 hours off (light cycle 6:00–19:00). For experiments involving tumor colonization with EcN, MC26 cells (BioHippo, 400156) were grown in DMEM media in T225 flasks. After trypsinization and resuspension in PBS + 0.1 mg ml^−1^ of DNAseI, 5 × 10^6^ MC26 cells were injected subcutaneously into the right flank of 6–8-week-old female Balb/cJ mice. Tumors were allowed to grow for 14 days (reaching sizes of 200–300 mm^3^) before injecting 10^8^ EcN cells suspended in PBS via the lateral tail vein. The day before injection of EcN, ibuprofen was added to the drinking water at 0.2 mg ml^−1^ to ameliorate side effects of EcN injections. To prepare the EcN for injection, the appropriate glycerol stocks were used to inoculate 2 ml of LB + 1% (w/v) glucose + 25 µg ml^−1^ of chloramphenicol, which was incubated at 37 °C and 250 r.p.m. overnight. The overnight culture (500 μl) was used to inoculate 50 ml of LB + 0.4% (w/v) glucose + 25 μg ml^−1^ of chloramphenicol in 250-ml baffled flasks, which was grown at 37 °C and 250 r.p.m. until reaching an OD_600_ of 0.3–0.6. This culture was pelleted, washed four times with PBS, resuspended in PBS at an OD_600_ of 0.625 and used for injection. Three days after injection of EcN, mice were injected intraperitoneally with 120 mg of L-arabinose to induce the EcN. Starting 24 hours after induction, ultrasound images of tumors were acquired as described in Supplementary Note 1. After imaging, 3.0-MPa acoustic pressure was applied throughout the tumor to collapse GVs, and mice were injected again intraperitoneally with 120 mg of L-arabinose. The next day, mice were imaged again with ultrasound for re-expression of GVs. The following day, all mice were euthanized, and tumors were excised, homogenized, serially diluted and plated on selective media (LB agar + 25 μg ml^−1^ of chloramphenicol) as well as on induction plates (LB agar + 25 μg ml^−1^ of chloramphenicol + 0.4% (w/v) glucose + 0.1% (w/v) L-arabinose). Colonies on plates with chloramphenicol were manually counted to quantify the levels of colonization, and colonies on induction plates were screened for a non-opaque mutant phenotype.

### Histology of tumors colonized by bacteria

Tumors were colonized with pBAD-bARG_Ser_-AxeTxe EcN following the same protocol as described above. The day after inducing GV expression with intraperitoneal injections of L-arabinose, BURST, xAM and B-mode images of tumors were acquired as described above. Shortly after imaging, mice were euthanized by sedation with isoflurane and cervical dislocation. Tumors were resected, placed in 10% buffered formalin for 48 hours and then washed and stored in 70% ethanol. Tumors were then cut in half along the approximate plane of imaging, placed in tissue cassettes and sent to the Translational Pathology Core Laboratory at the University of California, Los Angeles, which embedded samples in paraffin and performed hematoxylin and eosin (H&E) staining, immunohistochemistry and microscopy imaging. Immunohistochemistry was performed using Opal IHC kits (Akoya Biosciences) according to the manufacturer’s instructions. Tissue sections were incubated with either polyclonal rabbit anti-*E. coli* antibody (ViroStat, 1001) or non-reactive rabbit IgG isotype control antibody as a negative control. All sections were then incubated with an Opal 520 polymer anti-rabbit HRP antibody (Akoya Biosciences) and counterstained with DAPI. Sections were imaged in the appropriate fluorescence or bright-field channels using a high-throughput scanning system (Leica Aperio VERSA) with 40-µm resolution.

### Mammalian plasmid construction

Monocistronic plasmids were constructed using standard cloning techniques, including Gibson assembly and conventional restriction and ligation using primers listed in Supplementary Table 3. Coding sequences for the *A. flos-aquae* GV genes were codon-optimized and synthesized by Integrated DNA Technologies and subcloned into a pCMVSport backbone with a CMV promoter. *gvpA-*WPRE-hGH polyA was constructed by subcloning *gvpA* between PstI and MluI sites of pCMVSport vector with WPRE-hGH polyA.

Polycistronic mARG_Ana_ assembly factor genes *gvpNJKFGWV* were synthesized by Twist Bioscience in a pTwist-CMV vector. Emerald GFP was subcloned in-frame downstream of the *gvpNJKFGWV* open reading frame (ORF) via a P2A linker, and the entire *pNJKFGWV*-GFP ORF was subcloned into a pCMVSport backbone with WPRE-hGH-polyA elements using NEBuilder HiFi DNA Assembly (NEB). *gvpA-*IRES-EBFP2-WPRE-hGH polyA was constructed by Gibson assembly of a PCR-amplified IRES-EBFP2 fragment into the XbaI site of *gvpA-*WPRE-hGH polyA plasmid.

PiggyBac transposon plasmids were constructed by PCR-amplifying the region between the start codon of *gvpNJKFGWV* or *gvpA* and the end of the hGH polyA from the pCMVSport plasmids. The amplified regions were Gibson-assembled into the PiggyBac transposon backbone (System Biosciences) with a TRE3G promoter (Takara Bio) for doxycycline-inducible expression.

The lentiviral transfer plasmid with constitutively expressed tetracycline transactivator (pEF1α-rtTA-Antares-WPRE) was constructed as follows: pNCS-Antares was obtained from Addgene (74279), and P2A was added to the N-terminus of Antares with a primer overhang during PCR. This fragment was subcloned into the lentiviral transfer plasmid pEF1α-rtTA-WPRE between rtTA and WPRE in-frame with the rtTA ORF using NEBuilder HiFi DNA Assembly.

### HEK293T cell culture and transient transfection

HEK293T cells (American Type Culture Collection (ATCC), CLR-2316) were cultured in 24-well plates at 37 °C and 5% CO_2_ in a humidified incubator in 0.5 ml of DMEM (Corning, 10-013-CV) with 10% FBS (Gibco) and 1× penicillin–streptomycin until about 80% confluency before transfection. In brief, transient transfection mixtures were created by mixing of around 600 ng of plasmid mixture with polyethyleneimine (PEI-MAX, Polysciences) at 2.58 µg of polyethyleneimine per microgram of DNA. The mixture was incubated for 12 minutes at room temperature and added drop-wise to HEK293T cells. Media was changed after 12–16 hours and daily thereafter. For *gvpA* titration experiments, pUC19 plasmid DNA was used to keep the total amount of DNA constant.

After 3 days of expression, cells were dissociated using trypsin/EDTA, counted using disposable hemocytometers (Bulldog Bio) and centrifuged at 300*g* for 6 minutes at room temperature. Cells were resuspended with 1% low-melt agarose (GoldBio) in PBS at 40 °C at ~30 million cells per milliliter (Fig. 4b,f), ~15 million cells per milliliter (Fig. 4d) or ~7.5 million cells per milliliter (Supplementary Fig. 18c) before loading into wells of pre-formed phantoms consisting of 1% agarose (Bio-Rad) in PBS.

### Genomic integration and fluorescence-activated cell sorting

MDA-MB-231 (ATCC, HTB26), 3T3 (ATCC, CRL-1658) and HuH7 (JCRB0403) cells were cultured in DMEM (Corning, 10-013-CV) supplemented with 10% FBS (Gibco) and 1× penicillin–streptomycin at 37 °C and 5% CO_2_ in a humidified incubator unless noted otherwise. Cells were lentivirally transduced with pEF1α-rtTA-Antares-WPRE and sorted based on strong Antares fluorescence (Ex: 488 nm, Em: 610/20BP + 595LP) using BD FACSAria II for MDA-MB-231 cells and MACSQuant Tyto (B2 channel) for 3T3 and HuH7 cells. MDA-MB-231-rtTA-Antares cells were then electroporated in 20-µl format using 4D-Nucleofector using CH-125 protocol in SF buffer (Lonza) with 1 µg of PiggyBac transposon:transposase plasmid mixture (2:1 PB-gvpA:PB-gvpNV transposons, 285 ng of PiggyBac transposase). 3T3-rtTA-Antares cells were transfected with the same PiggyBac plasmid mixture using PEI-MAX in a 12-well format, and HuH7 cells were transfected using Lipofectamine 3000. Cells were expanded into surface-treated T75 flasks in TET-free media and were induced for 12 hours with 1 µg ml^−1^ of doxycycline before sorting for triple-positive cells (gated for Antares and then Emerald and EBFP2). The sorted cells were returned to DMEM with TET-free FBS (Takara Bio). MDA-MB-231-mARG_Ana_ was sorted twice. The first round of sorting was performed with permissive gates, and the enriched population was ~50% double-positive for Emerald and EBFP2 as analyzed with MACSQuant VYB (Miltenyi Biotec). This population was sorted again with stricter gates to ~95% purity. 3T3-mARG_Ana_ cells were sorted for strong Emerald and EBFP2 fluorescence using MACSQuant Tyto only once, yielding a population that was ~80% double-positive. HuH7 cells were sorted twice to ~91% purity using MACSQuant Tyto. Cells were expanded in TET-free media and frozen in Recovery Cell Culture Freezing Medium (Gibco) using Mr. Frosty cell freezing container (Nalgene) filled with isopropanol at −80˚C and then stored in liquid nitrogen vapor phase until use.

### TEM imaging of GVs expressed in mammalian cells

For TEM, cells were cultured in six-well plates in 2 ml of media. Then, 1 µg ml^−1^ of doxycycline was added to the wells at indicated times with daily media plus doxycycline changes thereafter until harvest. Cells were lysed by adding 400 µl of Solulyse-M (Genlantis) supplemented with 25 U ml^−1^ of Benzonase Nuclease (Novagen) directly to the six-well plates and incubating for 1 hour at 4 °C with agitation. The lysates were then transferred to 1.5-ml microcentrifuge tubes. Then, 800 µl of 10 mM HEPES (pH 7.5) was added to each tube, and lysates were centrifuged overnight at 300×*g* and 8° C. Next, 30 µl of the supernatant was collected from the surface on the side of the tube facing the center of the centrifuge rotor and transferred to a new tube. Then, 3 μl of each sample was loaded onto freshly glow-discharged (Pelco EasiGlow, 15 mA, 1 minute) formvar/carbon 200 mesh grids (Ted Pella) and blotted after 1 minute and then air-dried. The unstained grids were imaged on a FEI Tecnai T12 transmission electron microscope equipped with a Gatan Ultrascan CCD camera.

### Whole-animal fluorescence imaging of mARG_Ana_-expressing orthotopic tumors and tumor fluorescence microscopy

Tumor xenograft experiments were conducted in NSG mice aged 12 weeks and 6 days (Jackson Laboratory). To implement an orthotopic model of breast cancer, all the mice were female. MDA-MB-231-mARG_Ana_ cells were grown in T225 flasks in DMEM supplemented with 10% TET-free FBS and penicillin–streptomycin until confluency as described above. Cells were harvested by trypsinization with 6 ml of trypsin/EDTA for 6 minutes and quenched with fresh media. Cells were washed once in DMEM without antibiotics or FBS before pelleting by centrifugation at 300*×g*. Cell pellets were resuspended in a 1:1 mixture of ice-cold Matrigel (HC, GFR) (Corning, 354263) and PBS (Ca^2+^, Mg^2+^-free) at 30 million cells per milliliter. Then, 50-µl Matrigel suspensions were injected bilaterally into the 4th mammary fat pads at 1.5 million cells per tumor via subcutaneous injection. Twelve hours after tumor injection and every 12 hours thereafter (except the mornings of ultrasound imaging sessions), test mice were intraperitoneally injected with 150 µl of saline containing 150 µg of doxycycline for induction of GV expression. Control mice were not injected with doxycycline.

Mice were imaged using ultrasound as described in Supplementary Note 1. On the last day of ultrasound imaging, mice were anesthetized with 100 mg kg^−1^ of ketamine and 10 mg kg^−1^ of xylazine and whole-body imaged in supine position using ChemiDoc MP imaging system with Image Lab software (Bio-Rad). Fluorescence channels were set as follows: blue epi illumination with 530/28 filter for Emerald/GFP and 605/50 filter for Antares/CyOFP1. Images were processed and merged using the Fiji package of ImageJ.

After whole-body fluorescence imaging, mice were euthanized, and tumors were resected and placed in 10% formalin solution for 24 hours at 4 °C, after which they were transferred to PBS. Fixed tumors were embedded in 2% agarose in PBS and sectioned to 100-µm slices using a vibratome. Sections were stained with TO-PRO-3 nuclear stain, mounted using Prolong Glass (Invitrogen) and imaged using a Zeiss LSM 980 confocal microscope with ZEN Blue. Images were processed using the Fiji package of ImageJ. For micrographs of tumors from both induced and uninduced mice, the Emerald channel was capped between 0 and 25,497, EBFP2 channel between 0 and 17,233 and TO-PRO-3 channel between 5,945 and 53,136 for display.

### Reporting summary

Further information on research design is available in the Nature Portfolio Reporting Summary linked to this article.

## Online content

Any methods, additional references, Nature Portfolio reporting summaries, source data, extended data, supplementary information, acknowledgements, peer review information; details of author contributions and competing interests; and statements of data and code availability are available at10.1038/s41587-022-01581-y.

## Supplementary information

Supplementary Information Supplementary Notes 1 and 2 and Supplementary Figs. 1–11

Reporting Summary

Supplementary Tables 1–3

Supplementary Video 1 Supplementary Video 1: xAM/B-mode tomogram of an induced orthotopic tumor imaged on day 12. A representative tomogram of an orthotopic MDA-MB-231-mARGAna tumor imaged after 12 days of doxycycline induction. Each slice in the tomogram is separated by 100 µm.

Supplementary Video 2 Supplementary Video 2: xAM/B-mode tomogram of an induced chimeric tumor imaged on day 5. A representative tomogram of a chimeric MDA-MB-231-mARGAna tumor imaged after 5 days of doxycycline induction. Each slice in the tomogram is separated by 200 µm.

Supplementary Video 3 Supplementary Video 3: xAM/B-mode 3D reconstruction of an induced chimeric tumor imaged on day 5. 3D B-mode and xAM data were smoothened and converted to isosurfaces using MATLAB. Yellow 3D map represents B-mode density; blue 3D map represents xAM density.

Supplementary Video 4 Supplementary Video 4: Representative xAM/B-mode video of a chimeric tumor biopsy procedure sampling the xAM-positive region.

Supplementary Video 5 Supplementary Video 5: Representative xAM/B-mode video of a chimeric tumor biopsy procedure sampling the xAM-negative region.

Supplementary Data 3 Statistical Source Data

Supplementary Data 5 Statistical Source Data

Supplementary Data 6 Statistical Source Data

Supplementary Data 8 Statistical Source Data

## Data Availability

Plasmids will be made available through Addgene upon publication (https://www.addgene.org/Mikhail_Shapiro). All other materials and data are available from the corresponding author upon reasonable request. Genomic sequence information was downloaded from the NCBI sequence database via Batch Entrez (https://www.ncbi.nlm.nih.gov/sites/batchentrez). Source data are provided with this paper.

## Source data

Source Data Fig. 1 Statistical Source Data
Source Data Fig. 2 Statistical Source Data
Source Data Fig. 3 Statistical Source Data
Source Data Fig. 4 Statistical Source Data
Source Data Fig. 5 Statistical Source Data
Source Data Fig. 6 Statistical Source Data
Source Data Extended Data Fig. 1 Statistical Source Data
Source Data Extended Data Fig. 2 Statistical Source Data
Source Data Extended Data Fig. 3 Statistical Source Data
Source Data Extended Data Fig. 4 Statistical Source Data
Source Data Extended Data Fig. 5 Statistical Source Data
Source Data Extended Data Fig. 6 Statistical Source Data
Source Data Extended Data Fig. 7 Statistical Source Data
Source Data Extended Data Fig. 8 Statistical Source Data
Source Data Extended Data Fig. 9 Statistical Source Data

## Code availability

Ultrasound data acquisition and analysis code is available on the Shapiro laboratory GitHub at https://github.com/shapiro-lab.
