# Supplementary material for: Genomically mined acoustic reporter genes for real-time in vivo monitoring of tumors and tumor-homing bacteria
Source: Nat Biotechnol. 2023 Jan 2;41(7):919–31. doi: 10.1038/s41587-022-01581-y (PMC10344784; doi:10.1038/s41587-022-01581-y)
Supplement: Supplementary file 1 — Supplementary Notes 1 and 2 and Supplementary Figs. 1–11 [file 41587_2022_1581_MOESM1_ESM.pdf]

nature biotechnology

Article

https://doi.org/10.1038/s41587-022-01581-y

Genomically mined acoustic reporter genes for real-time in vivo monitoring of tumors and tumor-homing bacteria

In the format provided by the authors and unedited

SUPPLEMENTARY INFORMATION

Supplementary Note 1: Ultrasound imaging procedures

In vitro ultrasound imaging of bacteria expressing ARGs on solid media

Ultrasound imaging of bacterial patches was performed using a Verasonics Vantage programmable ultrasound scanning system and an L10-4v 128-element linear array transducer (Verasonics) with a center frequency of 6 MHz and an element pitch of 300 µm. Image acquisition was performed using a custom imaging script with a 64-ray-lines protocol and a synthetic aperture of 65 elements. The transmit waveform was set to a voltage of 50 V and a frequency of 10 MHz, with 1 waveform cycle and 67% intra-pulse duty cycle. In xAM mode, a custom sequence detailed previously[33] was used with an angle of 19.5°. RF data from 4 repeated acquisitions was coherently averaged prior to beamforming for each image plane.

Agar plates containing bacterial patches were coated with a thin layer of LB agar and immersed in PBS to allow acoustic coupling to the L10-4v transducer. The transducer was connected to a BiSlide computer-controlled 3D translatable stage (Velmex) and positioned above the plane of the plate at an angle of 15° from the vertical (to minimize specular reflection from the plastic dishes and agar) and a distance of 20 mm from the bacterial patches. The imaging sequence was applied sequentially to acquire image planes covering the full area of all plates. A custom script was used to automate the scan by controlling the motor stage in tandem with the ultrasound system, translating 0.5 mm in the azimuthal direction between rows and 19.5 mm in the lateral direction between columns. In the case of differential imaging scans, the full scan sequence was repeated after returning the motor stage to its origin and adjusting the voltage of the transducer.

For image processing and analysis, custom beamforming scripts were applied on-line to reconstruct image planes from the acquired RF data at each location. The intensity data for each plane was saved for off-line processing. All image planes were concatenated to form a 3D volume with all plates and colonies. A 2D image of the colonies was extracted from the 3D volume by taking the maximum intensity over a manually-defined depth range for all voxel columns. 2D differential images were obtained by subtracting the post-collapse 2D image from the pre-collapse 2D image. Bacterial patch intensities were then quantified from these 2D images. Sample ROIs were drawn around the center of each patch to avoid artefacts from the edges, and background ROIs were drawn around representative regions without patches. The signal-to-background ratio (SBR) was calculated as the mean pixel intensity of the sample ROI divided by the mean pixel intensity of the background.

Conversion to decibels (dB) was calculated as 20*log10(SBR). For display, images were normalized by dividing by the average background signal of all images being compared and setting the lower and upper limits of the colormaps to be the same, where the lower limit was equal to a constant A times the average background and the upper limit was equal to a constant B times the maximum pixel intensity out of all images being compared; images were then converted to dB. For xAM and differential xAM images of bacterial patches, A was set to 1 and B was set to 0.5.

In vitro ultrasound imaging of bacteria expressing ARGs suspended in agarose phantoms

To create phantoms for ultrasound imaging of bacteria from liquid cultures or suspended in PBS from patches on solid media, wells were cast with a custom 3D-printed mold using 1% (w/v) agarose in PBS, which was degassed by incubating at 65°C for at least 16 hours. Cultures or cell suspensions to be analyzed were diluted in ice-cold PBS to 2x the final desired cell concentration (calculated from the measured OD_{600}), incubated at 42°C for one minute, and mixed 1:1 with 1% (w/v) agarose in PBS at 42°C for a final concentration of 1x. This mixture was then loaded into the wells in duplicate and allowed to solidify. Care was taken not to introduce bubbles during this process. The phantoms were submerged in PBS, and ultrasound images were acquired using a Verasonics Vantage programmable ultrasound scanning system and an L22-14v 128-element linear array transducer with a center frequency of 18.5 MHz with 67%-6-dB bandwidth, an element pitch of 100 μm, an elevation focus of 8 mm, and an elevation aperture of 1.5 mm. The transducer was attached to a custom-made manual translation stage to move between samples. B-mode and xAM images were acquired using the same parameters as described previously:[71] the frequency and transmit focus were set to 15.625 MHz and 5 mm, respectively, and each image was an average of 50 accumulations. B-mode imaging was performed with a conventional 128-ray-lines protocol, where each ray line was a single pulse transmitted with an aperture of 40 elements. xAM imaging was performed using a custom sequence detailed previously[33] with an angle of 19.5° and an aperture of 65 elements. The transmitted pressure at the focus was calibrated using a Fibre-Optic Hydrophone (Precision Acoustics), and the peak positive pressure was used as the "acoustic pressure" in Fig. 3. BURST images were acquired as a series of pAM images as described previously,[7] except the focus was set to 6 mm, and the acoustic pressure was set to 0.15 MPa (1.6V) for the first 10 frames and 3.0 MPa (25V) for the last 46 frames.

To measure the xAM signal at varying acoustic pressures, an automated voltage ramp imaging script was used to acquire an xAM image at each voltage step (0.5 V increments from 2 to 25 V), immediately followed by a B-mode acquisition at a constant voltage of 1.6 V (0.15 MPa) before another xAM acquisition at the next voltage step; the voltage was held constant for 10 seconds at

each step before the image was saved. To measure the xAM and B-mode signals over time at various acoustic pressures, another script was used to automatically save an xAM or B-mode image every second while the voltage was automatically increased by 2 V approximately every 70 seconds. Each frame consisted of 64 ray lines, which took 180 µs each to acquire, giving a pulse repetition rate of 86.8 Hz. Based on these results, all subsequent *in vitro* xAM images of $bARG_{Ser}$-expressing EcN were acquired at 18V (1.29 MPa).

For the experiments in **Fig. S8** and **Extended Data Fig. 6**, a different transducer, an L22-14vX transducer, was used which had a different pressure-to-voltage calibration. Consequently, for ultrasound imaging of *S. Typhimurium*, xAM imaging was performed at 1.72 MPa (14V), unless otherwise noted, and BURST was performed using 0.16 MPa (1.6V) for the first 10 frames and 3.7 MPa (25V) for the final 46 frames.

xAM and B-mode image processing and analysis were performed as described above, except that custom beamforming scripts were applied off-line to reconstruct images from the saved RF data for each sample, no 3D reconstruction was performed as images captured at single locations, circular ROIs were drawn around sample and background regions (taking care to avoid bubbles) to calculate SBRs, and values of A=1.4 and B=0.5 were used to normalize images for display. BURST images were reconstructed using the signal template unmixing algorithm as described previously[10]; as above, circular ROIs were then drawn around sample and background regions to calculate SBRs and values of A=3 and B=1 were used to normalize images for display. Data were plotted using GraphPad Prism (v9.4.1).

In vivo bacterial ARG ultrasound imaging

Mice were anesthetized with 2% isoflurane and maintained at 37°C using a heating pad. Images were acquired using the L22-14v transducer attached to a manual translation stage described above. Any hair on or around the tumors was removed with Nair, and Aquasonic 100 ultrasound transmission gel was used to couple the transducer to the skin. Parabolic B-mode and parabolic AM (pAM) images were first acquired using a custom 128 ray line script. Each image was formed from 96 focused beam ray lines, each with a 32-element aperture and 6 mm focus. The transmit waveform was set to a voltage of 1.6V in B-mode or 8V in pAM and a frequency of 15.625 MHz, with 1 waveform cycle and 67% intra-pulse duty. In B-mode, each ray line was a single transmit with all 32 elements, and in pAM each ray line consisted of one transmit with all 32 elements followed by 2 transmits in which first the odd and then the even-numbered elements are silenced.[58] Subsequently, xAM images, additional B-mode images, and finally BURST images were acquired at the same location without moving the transducer using the same parameters as described above for the *in vitro* experiments

(e.g. 18V for xAM, 1.6V for B-mode, and 1.6V to 25V for BURST). At least two separate locations spaced at least 2 mm apart in each tumor were imaged with B-mode, pAM, and xAM. Ultrasound images of tumors were quantified as described above where the sample ROIs were drawn around the necrotic cores in the tumors and the background ROIs were drawn around regions in the gel above the mouse. Images were normalized and plotted on a dB scale as described above except the scaling factors were A=2.5 and B=1 for xAM and pAM and the corresponding B-mode tumor images, and A=10 and B=0.5 for BURST images.

***In vitro* ultrasound imaging of transient expression of GVs in HEK293T cells suspended in agarose phantoms**

Phantoms were imaged using L22-14v transducer (Verasonics) while submerged in PBS on top of an acoustic absorber pad. For BURST imaging, wells were centered around the 8 mm natural focus of the transducer and a BURST pulse sequence was applied in pAM acquisition mode as described above, except the focus was set to 8 mm, and the acoustic pressure was set to 0.26 MPa (1.6V) for the first 10 frames and 2.11 MPa (10V) for the remaining frames. The xAM voltage ramps and B-mode images were acquired concurrently using the same parameters as described above, except the transducer voltage was varied from 4 to 24V in steps of 0.5V for xAM, and 10 frames, each consisting of 15 accumulations, were acquired per voltage. The well depth and the B-mode transmit focus were set to 5 mm. All image quantification was performed as described above, where the sample ROIs were drawn inside the well and the background ROIs were drawn around an empty region in the agarose phantom for SBR calculation. All images were normalized and plotted on a dB scale as described above except the scaling factors were A=2 and B=0.5.

***In vitro* ultrasound imaging of MDA-MB-231 mARGAna cells suspended in agarose phantoms**

For all *in vitro* experiments, MDA-MB-231-mARG$_{Ana}$ cells were cultured in DMEM supplemented with 10% TET-free FBS and penicillin/streptomycin. For xAM imaging of MDA-MB-231-mARG$_{Ana}$ cells suspended in agarose phantoms, cells were cultured in 24-well plates in 0.5 mL media. For **Fig. 5h**, cells were seeded at 7,500 cells per well and induced with 1 µg/mL doxycycline after an overnight incubation and at subsequent days as indicated (5 replicates per condition), except for the uninduced control which was grown in a 10 cm dish without doxycycline. Media was changed daily thereafter until cell harvest. Cells were trypsinized with 100 µL Trypsin/EDTA for 6 minutes at 37˚C, after which the trypsin was quenched by addition of 900 µL media. The cell number was equalized between different days of expression at 140,000 cells and pelleted at 300g for 6 minutes. Cells were then resuspended in 20 µl 1% low-melt agarose (GoldBio) in PBS at 40˚C and loaded into the wells of

preformed 1% agarose (Bio-Rad) phantoms in PBS. Ultrasound images were acquired with L22-14v 128-element linear array transducer (Verasonics). xAM voltage ramp and B-mode images were acquired concurrently using the same parameters as described above (the transducer voltage was varied from 4 to 24V in steps of 0.5V for xAM and 10 frames, each consisting of 15 accumulations, were acquired per voltage. The B-mode transmit focus was set to 5 mm). Images taken at the voltage that produced peak xAM signal (9V, 0.54 MPa) were chosen for quantification. For **Fig. 4i,j**, cells were seeded at 66,666 cells per well and induced with the indicated doxycycline concentrations after an overnight incubation in TET-free media (4 replicates per doxycycline concentration). Cells were incubated for 4 days with daily media/doxycycline changes. Cells were harvested as above, and ~420,000 cells from each condition were loaded per agarose phantom well. xAM and B-mode images were acquired concurrently using the same parameters as described above except the transducer voltage was varied from 6V to 10V in steps of 0.5V for xAM and 120 frames, each consisting of 15 accumulations, were acquired per voltage (~75 seconds/voltage). The B-mode transmit focus was set to 6 mm. Images taken at 7.5V (0.42 MPa) were chosen for display and quantification in **Fig. 4i** (doxycycline response). For **Fig. 4k** and **Extended Data Fig. 8f-g,** cells were seeded in 10-cm dishes and induced as above for 4 days. Cells were harvested as above and resuspended at 60,000,000 cells/mL. 10-fold serial dilutions were performed with each cell line. Each cell dilution was mixed 1:1 with 2% low-melt agarose before loading into agarose phantom wells. Cells were imaged with an L22-14vX transducer at 5.5V (0.61 MPa) for xAM: the highest pressure that produced stable signal over a 30-second exposure and using 2V to 15V pAM BURST.

For imaging of MDA-MB-231 cells under thick liver tissue, cells were induced with doxycycline in T225 flasks for 4 days. Cells were harvested as above and resuspended at 30,000,000 cells/mL in 1% low-melt agarose in PBS prior to loading into agarose phantom wells. >1 cm beef liver section (99 Ranch Market) was overlaid on top of the agarose phantom and secured with needles. The phantom and liver were submerged in a PBS bath and the transducer was positioned 20 mm away from the interface between the liver and the agarose phantom. Ultrasound imaging was performed using a L10-4v linear array transducer (Verasonics) using the same parameters as above, except the xAM voltage was varied between 2V (0.078 MPa) and 30V (2.51 MPa). B-mode was acquired at 1.6V (0.25 MPa). Each voltage was held for 5 frames, each consisting of 15 accumulations.

All image quantification was performed as described above where the sample ROIs were drawn inside the well and the background ROIs were drawn around an empty region in the agarose phantom for SBR calculation. All Images were normalized and plotted on a dB scale as described above except the scaling factors were A=2 and B=0.5. The xAM/B-mode overlay was made with the

B-mode image as background. A binary alpha mask was applied to the xAM image, giving pixel values lower than 2x the average background a value of 0 and all values above this threshold a value of 1.

In vivo ultrasound imaging of mARGAna expressing orthotopic tumors

For ultrasound imaging, mice were depilated around the 4th mammary fat pads using Nair (Aloe Vera) for ultrasound coupling with Aquasonic 100 gel. Mice were anesthetized with 2.5% isoflurane and maintained at 37°C in supine position on a heating pad. The first imaging session (day 4) consisted of 8 induced tumors from 4 mice and 7 uninduced tumors from 4 mice. One of the uninduced mice died during the first imaging session, which resulted in two fewer uninduced control tumors for the remaining imaging sessions.

Ultrasound images were acquired with an L22-14v 128-element linear array transducer. xAM and B-mode images were acquired concurrently using the same parameters as described in the *in vitro* section above except the transducer voltage was held at constant 7.5V (0.42 MPa) for xAM and 3 frames, each consisting of 15 accumulations, were acquired per section. A motor stage was programed to move 100 μm per section for a total of 150 sections per tumor. The B-mode transmit focus was set to 6 mm. Ultrasound images of tumors were quantified as described above where the sample ROIs were drawn around the tumors and the background ROIs were drawn around regions in the gel above the mouse. Images were normalized and plotted on a dB scale as described above except the scaling factors were A=2 and B=0.5 for both xAM and the corresponding B-mode tumor images. The xAM volume quantification was performed by summing all pixel values from all sections in each tomogram between 2 mm and 10 mm in depth.

In vivo *ultrasound-guided biopsy of $mARG_{Ana}$-expressing chimeric tumors.*

Chimeric tumor biopsy experiments were conducted in female NCG mice aged 8-weeks (Charles River Laboratories). MDA-MB-231-$mARG_{Ana}$ and MDA-MB-231-rtTA-Antares cells were grown and harvested as above. Cell pellets were resuspended in a 1:1 mixture of ice-cold Matrigel (HC, GFR) (Corning 354263) and PBS (Ca^{2+}- and Mg^{2+}-free) at 30 million cells/mL. 100 μL Matrigel suspensions of MDA-MB-231-$mARG_{Ana}$ were injected bilaterally into the 4th mammary fat pads at 3 million cells per tumor lobe via subcutaneous injection. After 1 hr, additional 100 μL Matrigel suspensions of MDA-MB-231-rtTA-Antares were injected close to the edge of the blisters created by the first injections to create dual-lobed chimeric tumors with heterogeneous gene expression patterns. Mice were intraperitoneally injected with 150 μL of saline containing 150 μg of doxycycline for induction of GV expression starting 12 hours after tumor injection and then every 12 hours thereafter for 5 days.

Mice were prepared for ultrasound imaging as above. Ultrasound images were acquired with an L22-14vX 128-element linear array transducer. xAM and B-mode imaging was performed as above except the transducer voltage was held at 5.5V (0.481 MPa) for xAM and 1.6V (0.161 MPa) for B-mode and the motor stage was programed to move either 200 µm per section for whole-tumor scans or was held stationary for biopsy video acquisition. Image normalization and scaling was performed as above. xAM/B-mode overlay was made as above.

To perform a fine-needle aspiration biopsy, a 23G needle was fitted to a 3 mL Luer-lock syringe prefilled with PBS. The syringe was mounted on a 3D-printed holder attached to a manual translation stage. Each biopsy attempt consisted of positioning the ultrasound probe over a tumor and moving the needle into the field of view. The needle was then inserted into either the xAM-positive or xAM-negative region of the tumor, guided by live xAM and B-mode imaging. The needle was wiggled back-and-forth a couple times before pulling the syringe plunger to aspirate cells. The tumor sample was then ejected into a tube with PBS. The biopsy was repeated for attempts that did not produce a visible cell pellet. Each sample was treated with Trypsin/EDTA for 6 minutes at 37˚C, then quenched with fresh media.

Flow cytometry was performed with MACSQuant 10 (Miltenyi Biotec). GFP was measured with the B1 channel and Antares using B2. All biopsy attempts for a given tumor/sampling condition were analyzed separately, but their resulting FCS data files were concatenated. Data analysis was performed in FlowJo. For quantification of biopsy samples, each population was first gated for Antares-positive cells to exclude endogenous mouse cells. Antares-positive cells were then gated based on FSC/SSC and single cells were gated using FSC-A vs FSC-W. The resulting populations contained on average 6947 cells with a SD of 6960 cells and range between 72 and 21958 cells. %GFP-positive ($mARG_{Ana}$-positive) was assessed based on these resulting populations.

Supplementary Note 2: Parameters for fitting xAM ultrasound signal versus time data to an exponential decay function

Data from Fig. S8b were first converted to linear scaling (not decibel) and then normalized so that at t = 0, the xAM signal y was equal to 1. The normalized data were then fit to the equation y = A*exp(-

k*t) + B, where the parameter k is the rate of exponential decay, B corresponds to the fraction of signal remaining at $t = \infty$, and A corresponds to the fraction of signal lost between $t = 0$ and $t = \infty$. As the applied acoustic pressure was increased, the fraction of signal lost A increased and the fraction of signal remaining B decreased, while the rate of decay remained relatively constant with a slight increase. See Fig. S8d for a plot of the fitted curves overlayed with the data.

**Pressure (MPa)**	**A**		**k (min^{-1})**		**B**		**Goodness of fit (R^2)**
	Value	SEM	Value	SEM	Value	SEM	
**1.10**	0.113	0.009	5.085	0.873	0.857	0.004	0.327
**1.41**	0.191	0.008	5.777	0.483	0.764	0.003	0.658
**1.72**	0.259	0.012	5.703	0.527	0.692	0.004	0.613
**1.97**	0.307	0.011	5.499	0.374	0.620	0.004	0.748
**2.23**	0.346	0.009	6.622	0.323	0.574	0.003	0.843

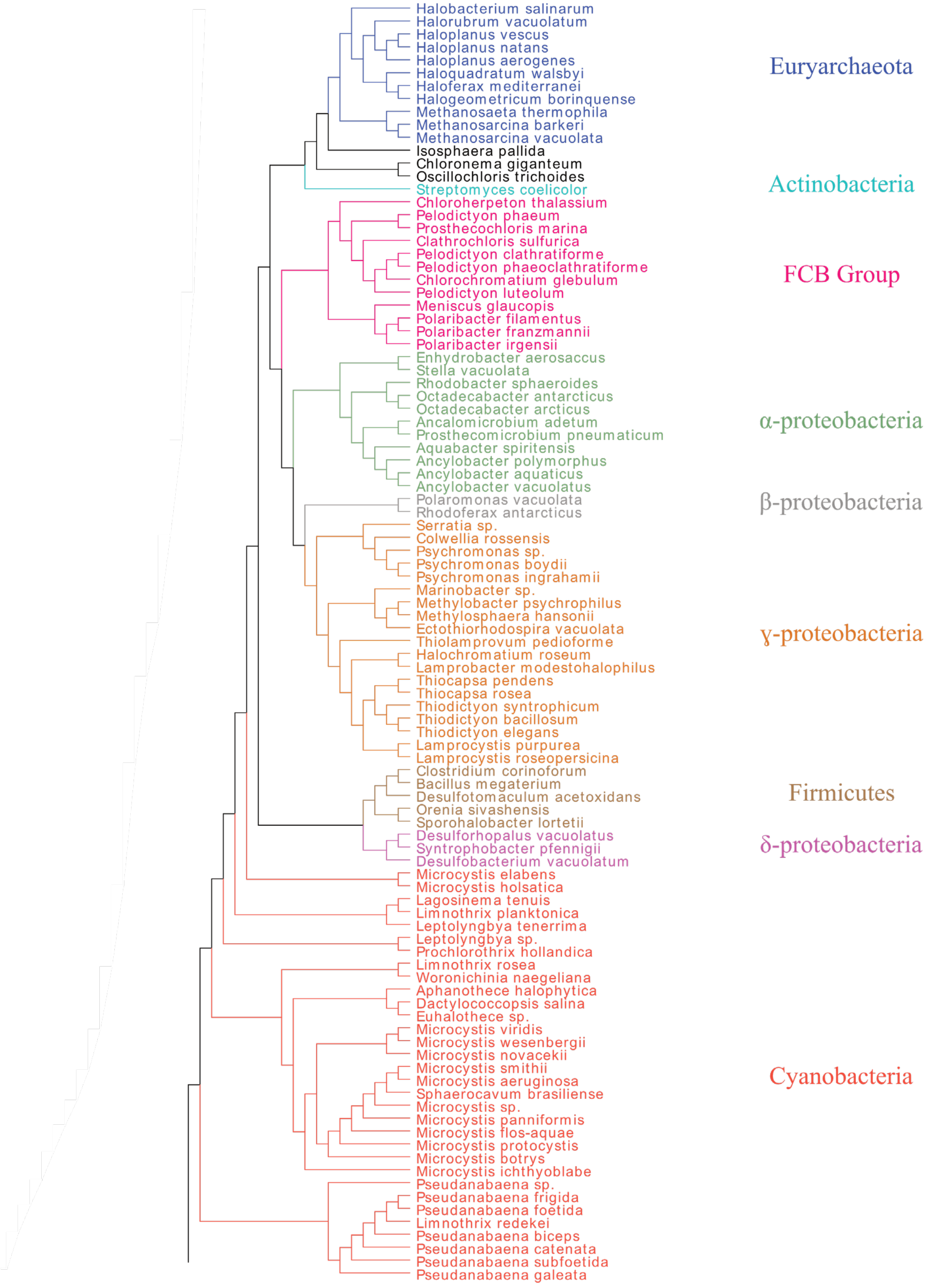
Halobacterium salinarum
Halorubrum vacuolatum
Haloplanus vescus
Haloplanus natans
Haloplanus aerogenes
Haloquadratum walsbyi
Haloferax mediterranei
Halogeometricum borinquense
Methanosaeta thermophila
Methanosarcina barkeri
Methanosarcina vacuolata
Isosphaera pallida
Chloronema giganteum
Oscillochloris trichoides
Streptomyces coelicolor
Chloroherpeton thalassium
Pelodictyon phaeum
Prosthecochloris marina
Clathrochloris sulfurica
Pelodictyon clathratiforme
Pelodictyon phaeoclathratiforme
Chlorochromatium glebulum
Pelodictyon luteolum
Meniscus glaucopis
Polaribacter filamentus
Polaribacter franzmannii
Polaribacter irgensii
Enhydrobacter aerosaccus
Stella vacuolata
Rhodobacter sphaeroides
Octadecabacter antarcticus
Octadecabacter arcticus
Ancalomicrobium adetum
Prosthecomicrobium pneumaticum
Aquabacter spiritensis
Ancylobacter polymorphus
Ancylobacter aquaticus
Ancylobacter vacuolatus
Polaromonas vacuolata
Rhodoferax antarcticus
Serratia sp.
Colwellia rossensis
Psychromonas sp.
Psychromonas boydii
Psychromonas ingrahamii
Marinobacter sp.
Methylobacter psychrophilus
Methylosphaera hansonii
Ectothiorhodospira vacuolata
Thiolamprovum pedioforme
Halochromatium roseum
Lamprobacter modestohalophilus
Thiocapsa pendens
Thiocapsa rosea
Thiodictyon syntrophicum
Thiodictyon bacillosum
Thiodictyon elegans
Lamprocystis purpurea
Lamprocystis roseopersicina
Clostridium corinoforum
Bacillus megaterium
Desulfotomaculum acetoxidans
Orenia sivashensis
Sporohalobacter lortetii
Desulforhopalus vacuolatus
Syntrophobacter pfennigii
Desulfobacterium vacuolatum
Microcystis elabens
Microcystis holsatica
Lagosinema tenuis
Limnothrix planktonica
Leptolyngbya tenerrima
Leptolyngbya sp.
Prochlorothrix hollandica
Limnothrix rosea
Woronichinia naegeliana
Aphanothece halophytica
Dactylococcopsis salina
Euhalothece sp.
Microcystis viridis
Microcystis wesenbergii
Microcystis novacekii
Microcystis smithii
Microcystis aeruginosa
Sphaerocavum brasiliense
Microcystis sp.
Microcystis panniformis
Microcystis flos-aquae
Microcystis protocystis
Microcystis botrys
Microcystis ichthyoblabe
Pseudanabaena sp.
Pseudanabaena frigida
Pseudanabaena foetida
Limnothrix redekei
Pseudanabaena biceps
Pseudanabaena catenata
Pseudanabaena subfoetida
Pseudanabaena galeata
Euryarchaeota
Actinobacteria
FCB Group
α-proteobacteria
β-proteobacteria
γ-proteobacteria
Firmicutes
δ-proteobacteria
Cyanobacteria

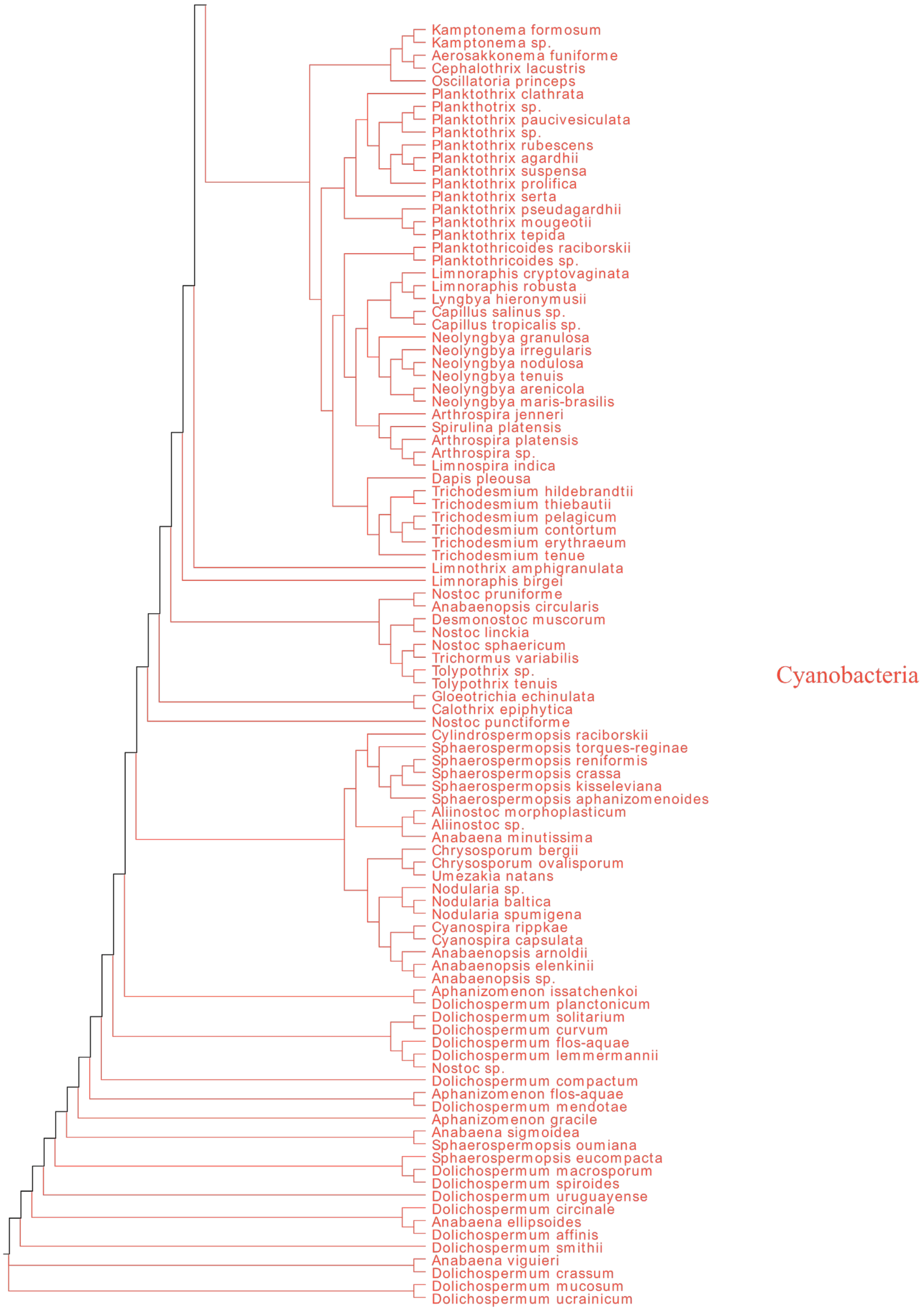

Supplementary Figure 1: 16S phylogenetic tree of all reported GV-producing organisms. Colors indicate groupings of phylogenetically similar organisms. Organisms from which GV genes were tested in *E. coli* are shown in **Fig. 1a**.

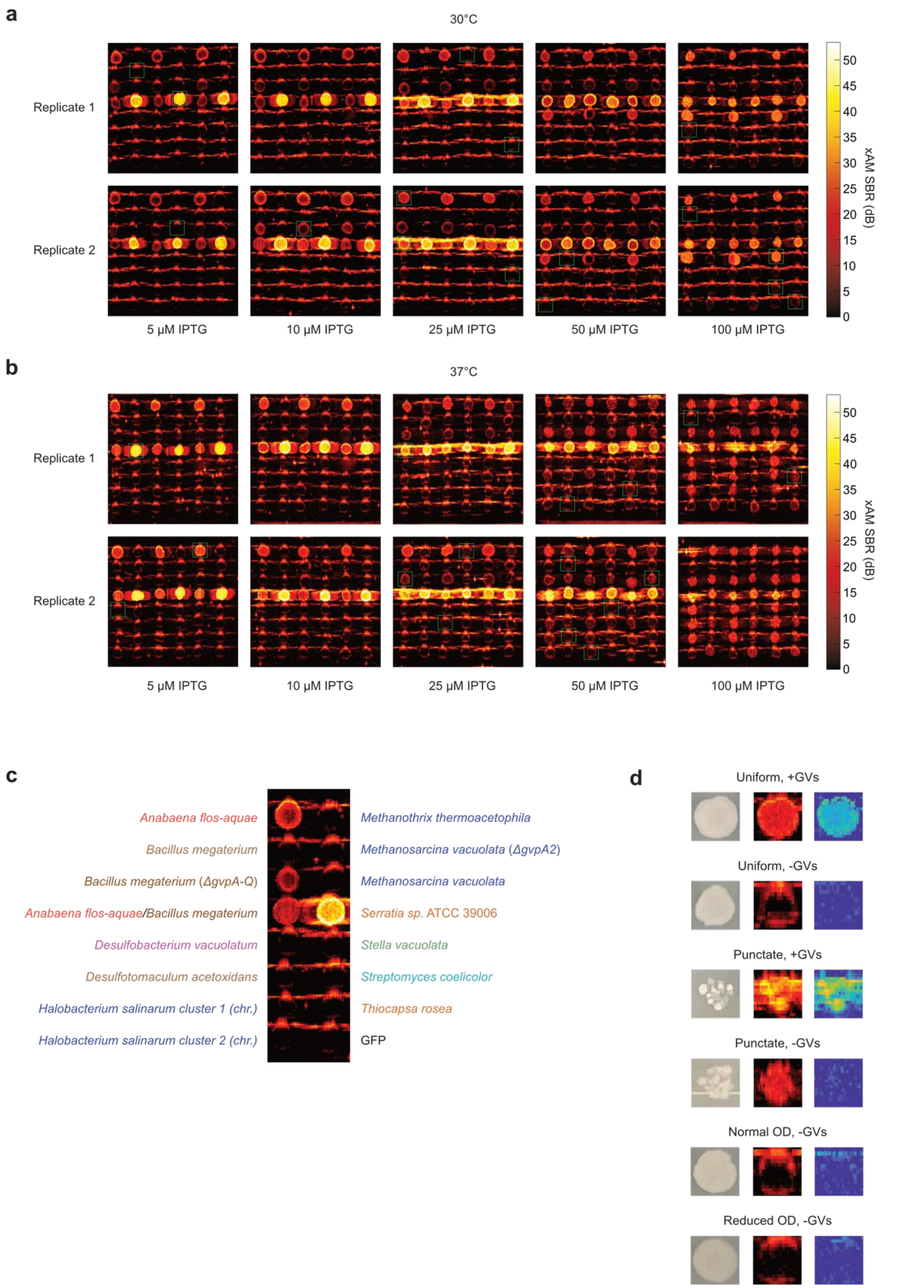
a
30°C
Replicate 1
Replicate 2
5 µM IPTG
10 µM IPTG
25 µM IPTG
50 µM IPTG
100 µM IPTG
xAM SBR (dB)
50
45
40
35
30
25
20
15
10
5
0
b
37°C
Replicate 1
Replicate 2
5 µM IPTG
10 µM IPTG
25 µM IPTG
50 µM IPTG
100 µM IPTG
xAM SBR (dB)
c
Anabaena flos-aquae
Bacillus megaterium
Bacillus megaterium (ΔgvpA-Q)
Anabaena flos-aquae/Bacillus megaterium
Desulfobacterium vacuolatum
Desulfotomaculum acetoxidans
Halobacterium salinarum cluster 1 (chr.)
Halobacterium salinarum cluster 2 (chr.)
Methanothrix thermoacetophila
Methanosarcina vacuolata (ΔgvpA2)
Methanosarcina vacuolata
Serratia sp. ATCC 39006
Stella vacuolata
Streptomyces coelicolor
Thiocapsa rosea
GFP
d
Uniform, +GVs
Uniform, -GVs
Punctate, +GVs
Punctate, -GVs
Normal OD, -GVs
Reduced OD, -GVs

Supplementary Figure 2: Optimization of expression conditions for all tested clusters in BL21(DE3) *E. coli.* (**a-b**) xAM images of bacterial patches expressing each GV cluster at varying inducer concentrations and temperatures. Green boxes indicate the patches shown in **Extended Data Fig. 1a**. The IPTG concentration selected was the one that resulted in the highest xAM pre-minus-post-collapse difference signal (**Fig. S5**) while not creating toxicity, as determined by whether the patch was uniform or punctate (**Fig. S5a-b**). Some of the IPTG concentrations that led to toxicity also created significant xAM signal, but this signal did not originate from GVs, as indicated by the lack of xAM pre-minus-post-collapse signal difference (**Fig. S4**). Further, there were some IPTG concentrations for certain genotypes that created significant xAM signal but no xAM pre-minus-post-collapse signal difference, and no visible toxicity (e.g., *Streptomyces coelicolor*, *Thiocapsa rosea*, and GFP at 37˚C, 100 µM IPTG). This discrepancy was likely caused by subtle toxicity that is not apparent in optical images, but altered the texture of the patch enough to be detectable by US. (**c**) Key for genotypes tested in (a-b), with this pattern repeated in three pairs of columns replicated on each plate. (**d**) Examples of the effects of toxic genotypes on bacterial patches, and of artifacts that can appear in bacterial patch scan images. Bacteria themselves can produce significant xAM signal (especially when present in extremely high concentrations, as they are in the confluent patches imaged here), which can be seen in the forms of rings around all patches, regardless of GV expression status. Further, expression of toxic proteins (or of large amounts of otherwise non-toxic proteins, such as GFP) can interfere with bacterial growth; in extreme cases this results in significant cell death and a punctate appearance, and in less extreme cases it simply reduces the optical density of patches. GV expression can increase the optical density of patches, but only at high levels of GV expression. Punctate patches produce considerably more xAM signal than uniform ones, even in the absence of GV expression. The xAM pre-minus-post-collapse difference can be used to qualitatively determine if a patch produces GVs, but because collapse is incomplete in some cases, it is not an ideal method for quantitatively comparing genotypes.

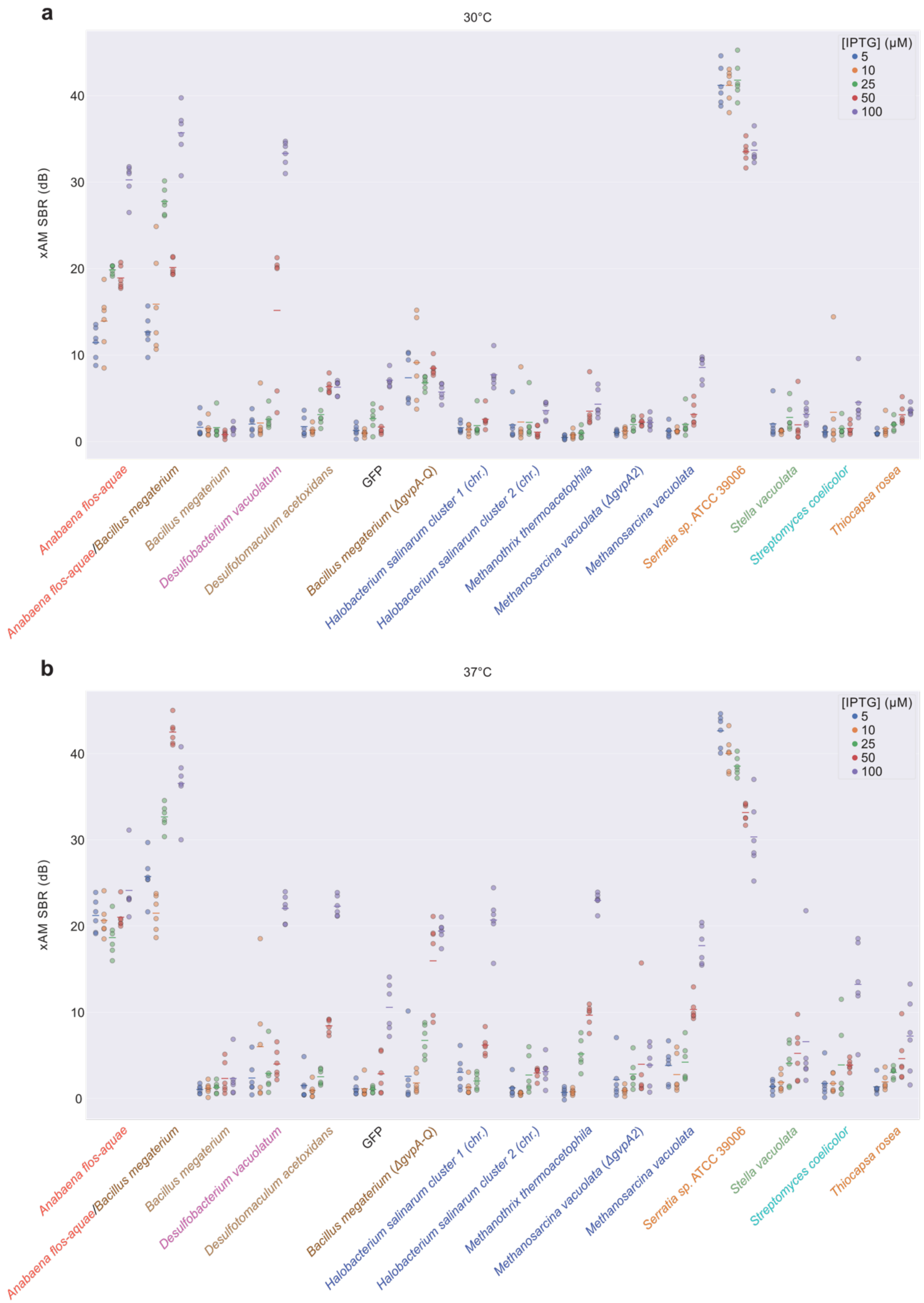

Supplementary Figure 3: Quantification of ultrasound signal for all samples shown in Fig. S2a-b. (**a-b**) xAM SBR of the patches at 30°C (a) and 37°C (b) shown in **Fig. S2a-b** (n=6; lines represent the mean).

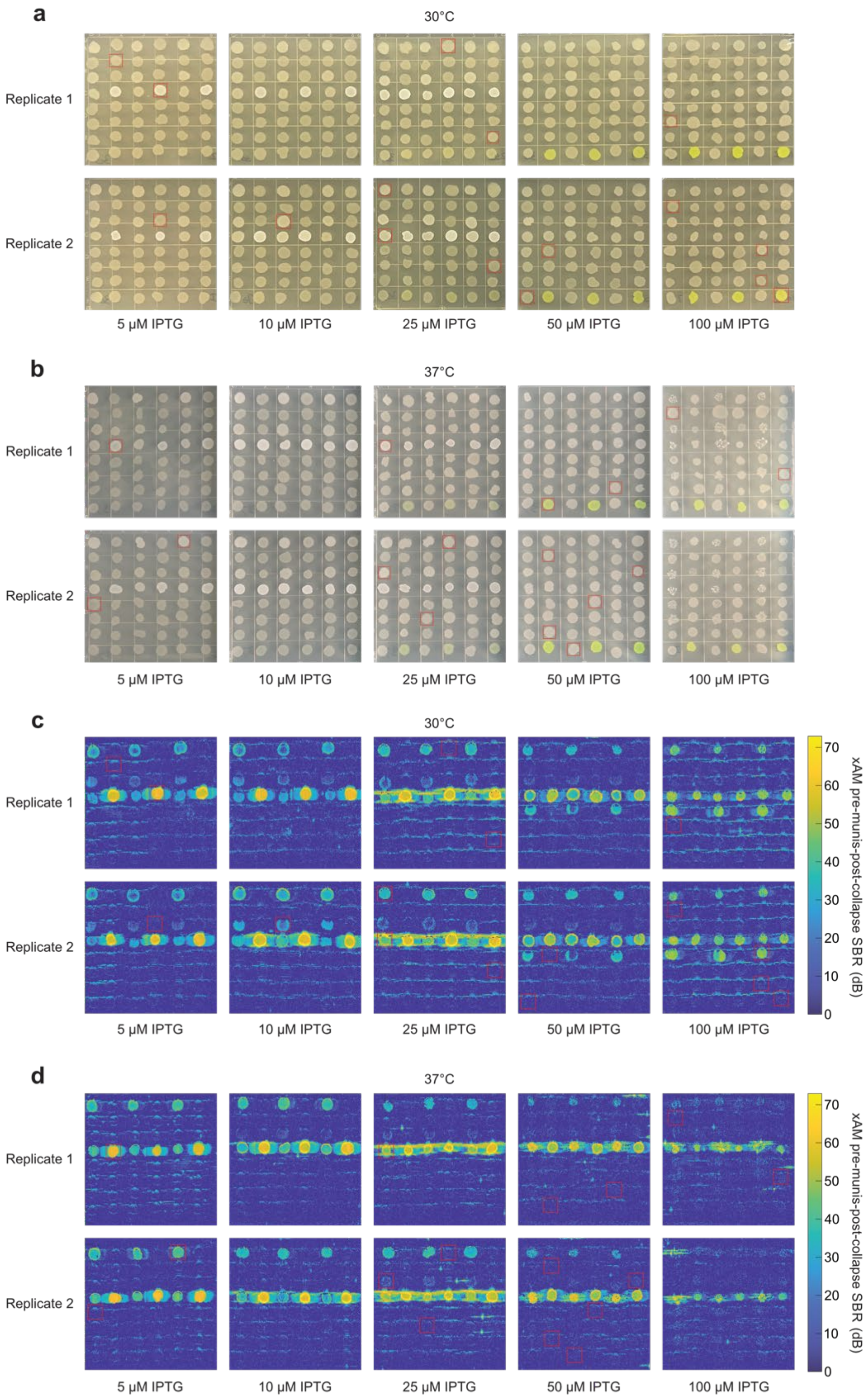

Supplementary Figure 4: Optical and xAM pre-minus-post-collapse difference images of all samples shown in Fig. S2. (**a-b**) Optical images of patches at 30°C (a) and 37°C (b) shown in **Fig. S2a-b**. (**c-d**) xAM pre-minus-post-collapse difference patches of samples at 30°C (a) and 37°C (b) shown in **Fig. S2a-b**. Red boxes indicate the patches shown in **Extended Data Fig. 1b-c**.

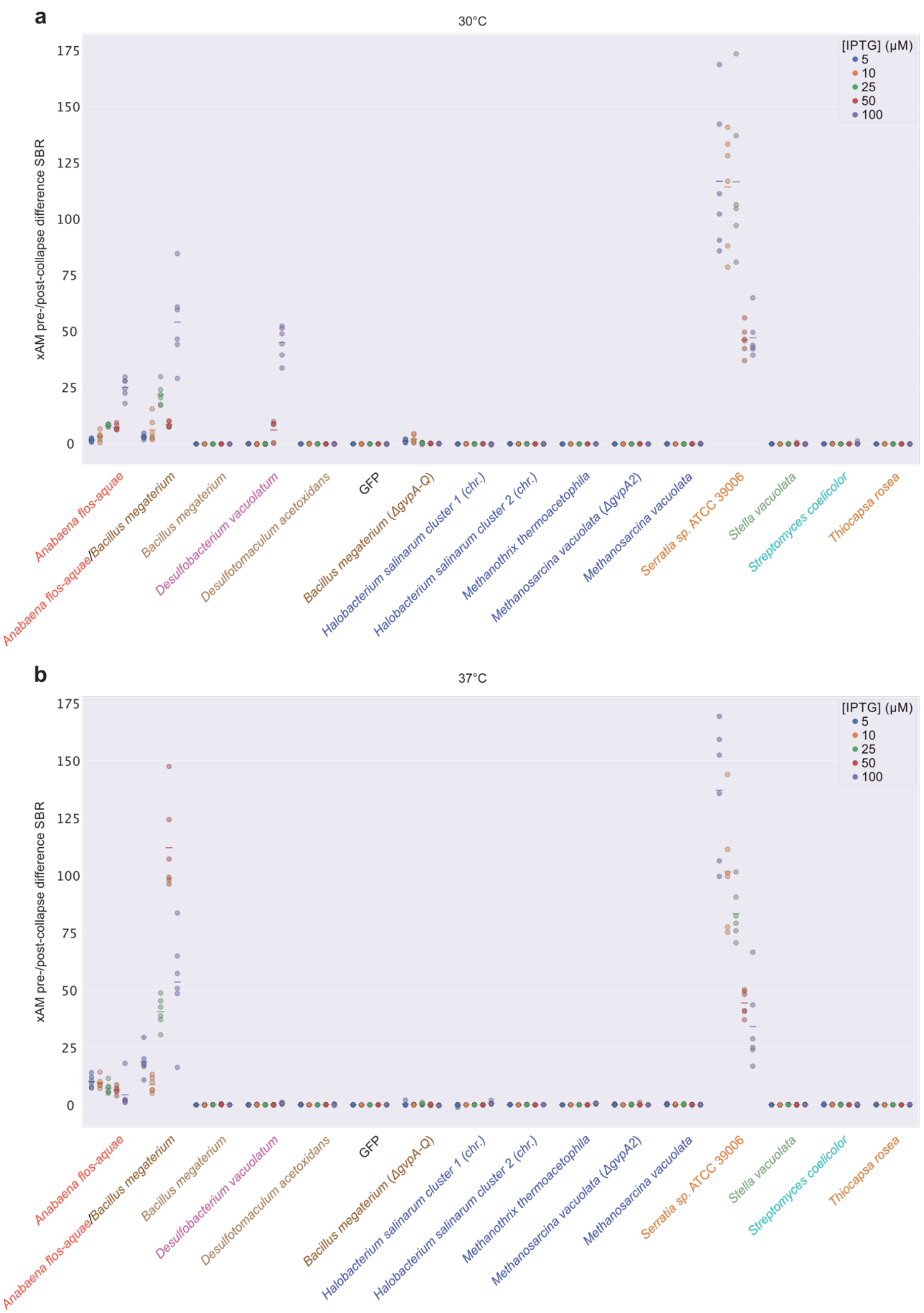

Supplementary Figure 5: Quantification of ultrasound signal for samples shown in Fig. S4c-d. (**a-b**) xAM SBR for the patches at 30°C (a) and 37°C (b) shown in **Fig. S4c-d** (n=6; lines represent the mean).

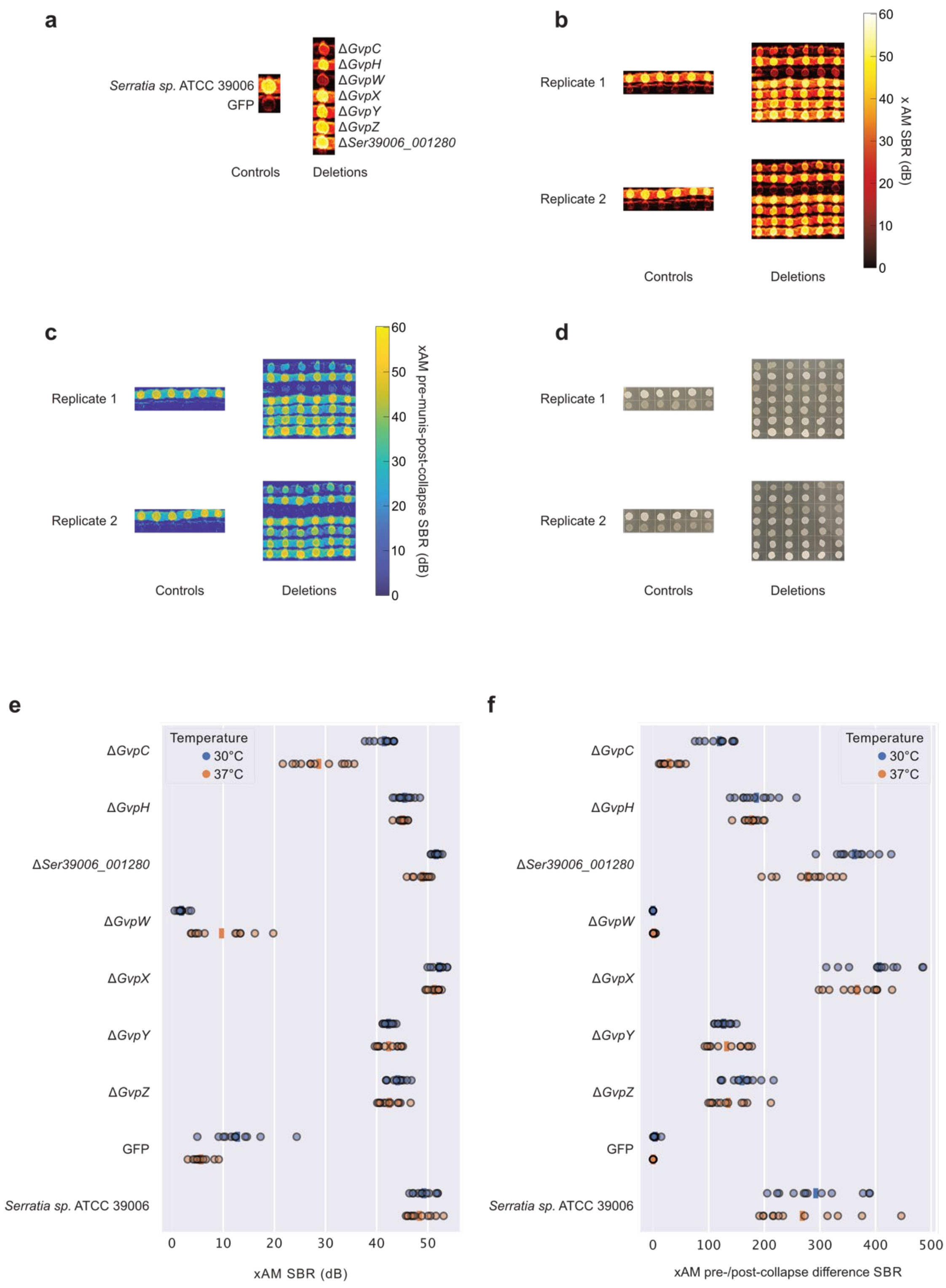

Supplementary Figure 6: Effects of single-gene deletions on GV expression by the *Serratia* cluster. (**a**) Key for genotypes tested, repeated in 6 replicate columns on each plate. (**b-d**) xAM images (b), pre-minus-post-collapse xAM images (c), and optical images (d) of bacterial patches expressing single-gene deletions of the Serratia cluster. (**e-f**) Quantification of the xAM images (e) and pre-minus-post-

collapse xAM images (f) shown in (b-c) (n=12 biologically independent samples examined in one experiment).

a

			Non-white mutants / total colonies screened			
**Condition**	**Replicate**	**Starter**	**Day 1**	**Day 2**	**Day 3**	**Day 5**
+chlor	#1	0/226	0/68	0/69	0/60	0/40
+chlor	#2		0/55	0/47	0/355	0/54
+chlor	#3		0/48	0/39	0/98	0/42
+chlor	#4		0/56	0/21	0/49	0/233
-chlor	#1		0/39	0/54	0/70	0/64
-chlor	#2		0/28	0/46	0/65	0/46
-chlor	#3		0/47	0/57	0/75	0/52
-chlor	#4		0/36	0/57	0/57	0/38
-chlor +ara	#1		0/2	0/68	0/53	0/44
-chlor +ara	#2		0/2	0/62	2/86	0/68
-chlor +ara	#3		ND*	0/67	3/102	6/70
-chlor +ara	#4		0/1	0/76	0/34	0/56

*ND: no data because there were no colonies

b

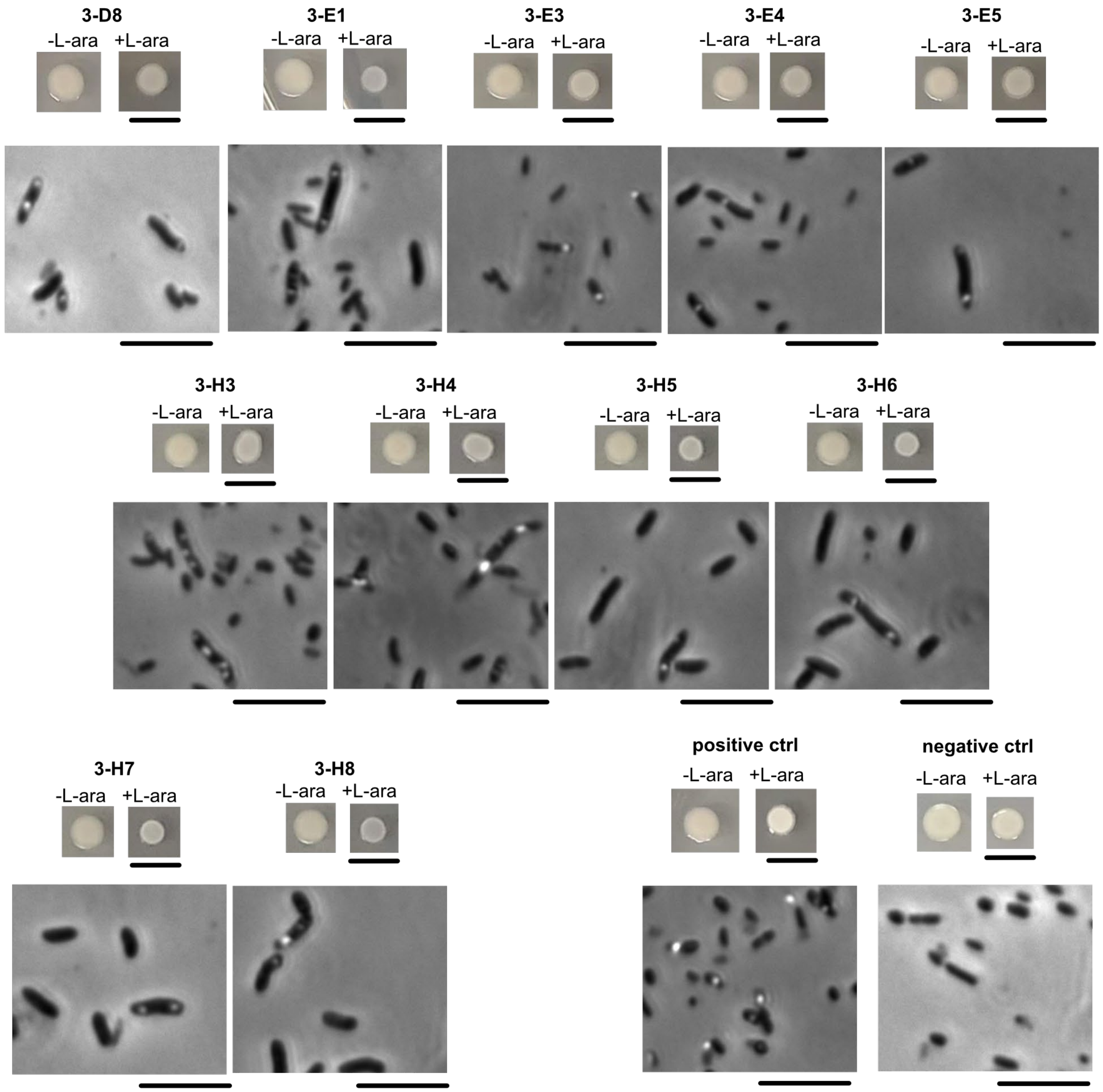

Supplementary Figure 7: Quantification and characterization of EcN mutants deficient in $bARG_{Ser}$ expression isolated from daily subculturing *in vitro*. (**a**) Numbers of non-white mutant colonies and total colonies screened on plates with 0.1% (w/v) L-arabinose from daily sub-culturing into LB media with 25 µg/mL chloramphenicol (+chlor), without chloramphenicol (-chlor), or without chloramphenicol and with 0.1% (w/v) L-arabinose (-chlor +L-ara) using pBAD-$bARG_{Ser}$-AxeTxe EcN. Cultures where mutants were found are indicated in red. (**b**) Optical images of patches (top rows) on fresh plates with 0.1% (w/v) L-arabinose (+L-ara) and without L-arabinose (-L-ara), and phase contrast microscopy images (bottom rows) from the 11 mutant colonies in (a). Mutants 3-D3 and 3-E1 were from the culture -chlor +ara, replicate #2, day 3; mutants 3-E3, 3-E4, and 3-E5 were from the culture -chlor +ara, replicate #3, day 3; and mutants 3-H3 through 3-H8 were from the culture -chlor +ara, replicate #3, day 5. The positive and negative controls were wild-type pBAD-$bARG_{Ser}$-AxeTxe EcN and pBAD-FP-AxeTxe EcN, respectively. Scale bars are 1 cm for images of patches and 10 µm for microscopy images.

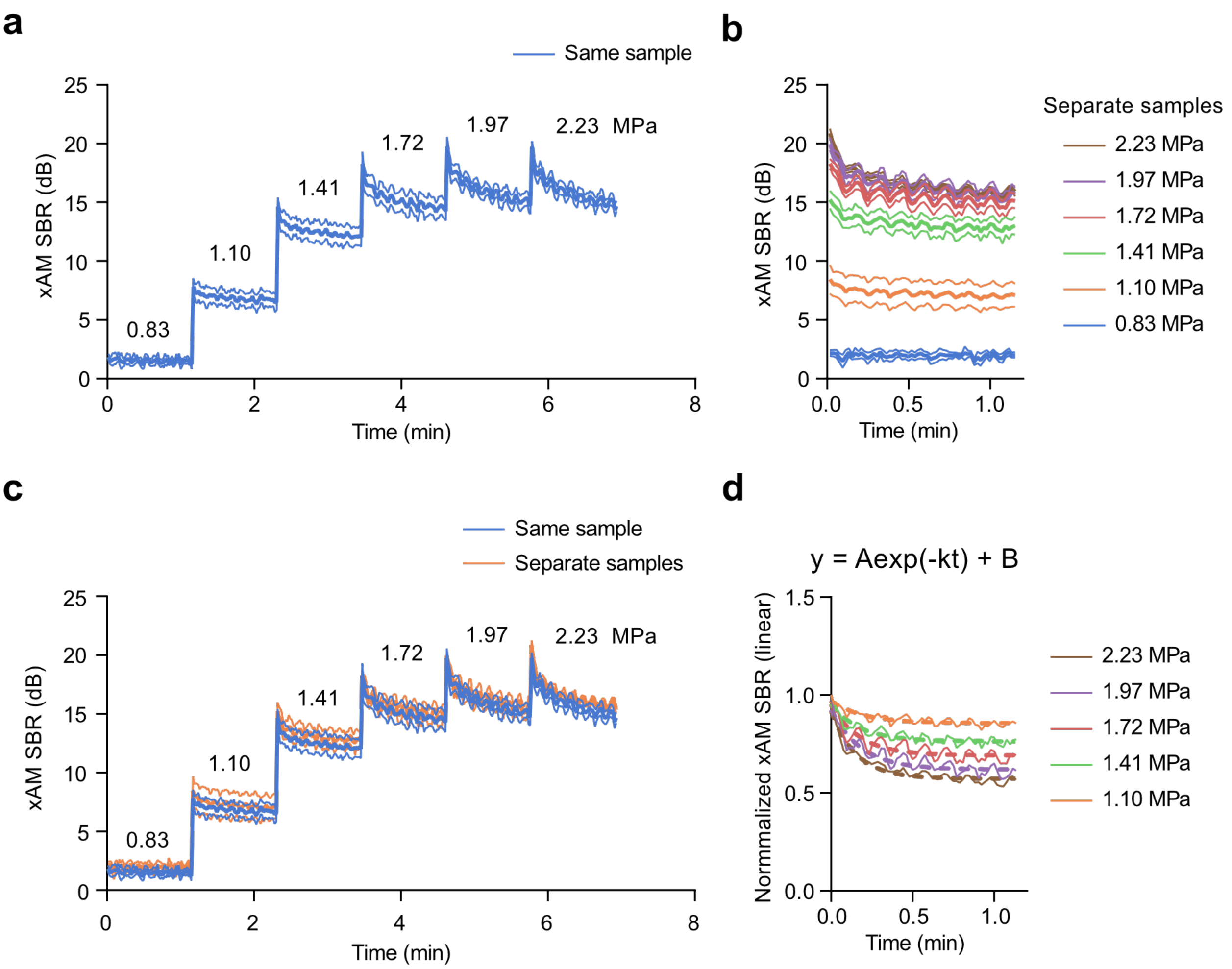

Supplementary Figure 8: xAM ultrasound signal versus time at varying acoustic pressures applied sequentially to the same sample versus separate samples. (**a-b**) xAM SBR of bARG$_{Ser}$-expressing EcN measured over time at various acoustic pressures. In (a), samples were subjected sequentially to 6 increasing acoustic pressures for approximately 70 sec each, whereas in (b) separate samples subjected to only one acoustic pressure for approximately 70 sec. (**c**) Overlay of xAM SBR curves for separate samples from (b) onto the curves for samples subjected to all pressures from (a). There is no difference between these curves, indicating that the xAM SBR measured at a certain pressure was not significantly affected by collapse at a previously applied pressure. For (a-c), pBAD-bARG$_{Ser}$-AxeTxe EcN were induced with 0.1% (w/v) L-arabinose for 24 hours at 37°C in liquid culture, and were then normalized to 10^9 cells/mL in agarose phantoms for ultrasound imaging. Bold lines represent the mean and thin lines represent ± standard deviation; n=3 biological replicates, each with 2 technical replicates. Imaging was performed with an L22-v14X transducer, so the values for pressure and xAM SBR do not exactly match those in **Fig. 2d-f** where an L22-v14 transducer was used. (d) Normalized and linear-scaled data from (b) shown by thin lines which were fit to the exponential decay function y = Aexp(-kt) + B shown by thick dotted lines. See **Supplementary Note 2** for the fitted parameter values.

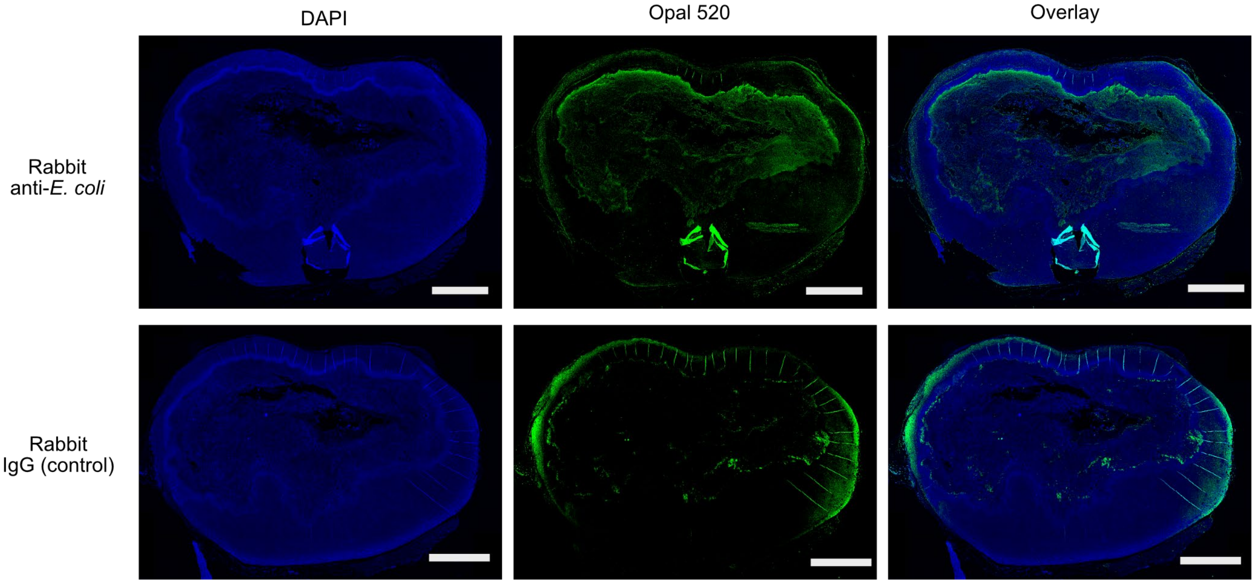

Supplementary Figure 9: Histology of MC26 tumor colonized with bARG$_{Ser}$-expressing EcN. Fluorescent images of tissue sections after ultrasound imaging on day 19 (see **Fig. 3a**). Sections were incubated with either polyclonal rabbit anti-*E. coli* antibodies (top row) or non-reactive rabbit IgG isotype control antibody (bottom row) as a negative control. All sections were then incubated with an Opal 520 polymer anti-rabbit HRP antibody (Akoya biosciences) and counterstained with DAPI. The EcN are visible in the necrotic core in the Opal 520 channel (top middle panel); the edges of the tissue exhibit a high degree of background staining (bottom middle panel). Histology was not repeated across multiple tumors because the other tumor tissues were instead homogenized and analyzed for colony forming units.

a

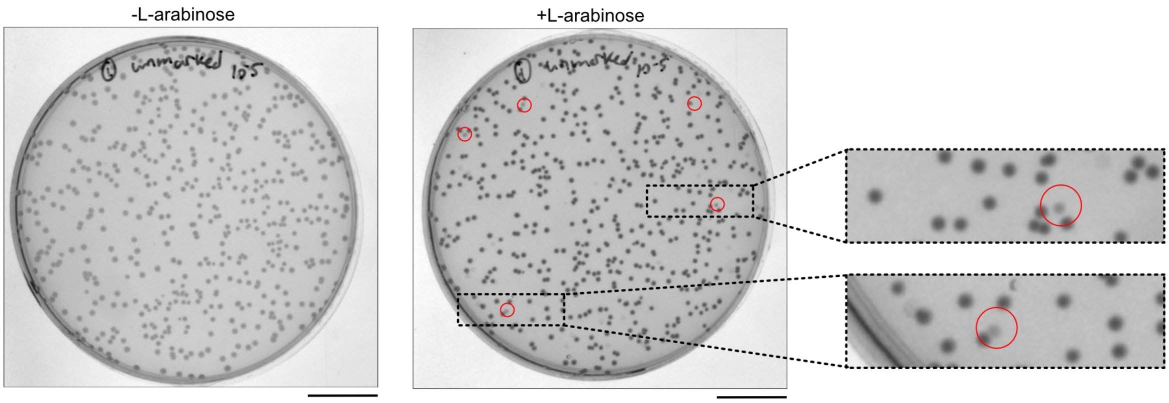

b

Mouse	Condition	Non-white mutants / total colonies screened
#1	Induced	0/687
#2	Induced	2/807
#3	Induced	0/341
#4	Induced	0/478
#5	Induced	5/585
#6	Uninduced	0/660
#7	Uninduced	0/567
#8	Uninduced	0/553
#9	Uninduced	0/556
#10	Uninduced	0/863
Total		7/6097 ~ 0.1%

c

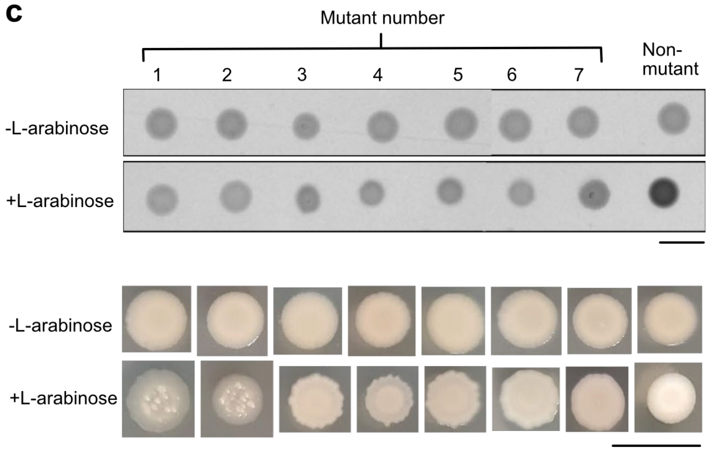

Supplementary Figure 10: Screening for EcN mutants defective in bARG$_{Ser}$ expression isolated from colonized tumors. (**a**) White light transmission images of plates with 0.1% (w/v) L-arabinose and without L-arabinose from plating a tumor (from mouse #5 in (b)) colonized by bARG$_{Ser}$-expressing EcN. Mutant colonies on the +L-arabinose plate appear lighter (more translucent) than wild-type opaque colonies and are indicated by red circles. (**b**) Numbers of non-white mutant colonies and total colonies screened on plates with 0.1% (w/v) L-arabinose for the ten mice injected with pBAD-bARG$_{Ser}$-AxeTxe EcN. **c**, White light transmission images (top) and photographs (bottom) of patches on fresh plates with 0.1% (w/v) L-arabinose and without L-arabinose made from the seven translucent mutant colonies in red in (b) and an opaque non-mutant colony as a control. Mutants 1-2 were from mouse #2 and mutants 3-7 were from mouse #5. Scale bars are 2 cm in (a) and 1 cm in (c).

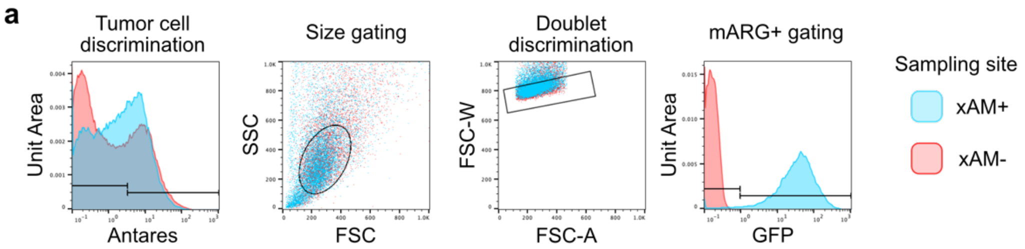

Supplementary Figure 11: Flow cytometric gating strategy for chimeric tumor biopsy sample analyses. Events were first gated based on Antares expression to exclude endogenous mouse cells. Antares-positive cells were then gated by size. Single cells were gated based on FSC-W vs FSC-A plot. GFP-positive (mARG$_{\text{Ana}}$-positive) cells were gated from the resulting histograms.
