# Supplementary material for: Genomically mined acoustic reporter genes for real-time in vivo monitoring of tumors and tumor-homing bacteria
Source: Nat Biotechnol. 2023 Jan 2;41(7):919–31. doi: 10.1038/s41587-022-01581-y (PMC10344784; doi:10.1038/s41587-022-01581-y)
Supplement: Supplementary file 2 — Reporting Summary [file 41587_2022_1581_MOESM2_ESM.pdf]

nature portfolio

Corresponding author(s): Mikhail G. Shapiro

Last updated by author(s): Oct 18, 2022

Reporting Summary

Nature Portfolio wishes to improve the reproducibility of the work that we publish. This form provides structure for consistency and transparency in reporting. For further information on Nature Portfolio policies, see our Editorial Policies and the Editorial Policy Checklist.

Statistics

For all statistical analyses, confirm that the following items are present in the figure legend, table legend, main text, or Methods section.

n/a	Confirmed	
☐	☒	The exact sample size (n) for each experimental group/condition, given as a discrete number and unit of measurement
☐	☒	A statement on whether measurements were taken from distinct samples or whether the same sample was measured repeatedly
☐	☒	The statistical test(s) used AND whether they are one- or two-sided<br>*Only common tests should be described solely by name; describe more complex techniques in the Methods section.*
☒	☐	A description of all covariates tested
☒	☐	A description of any assumptions or corrections, such as tests of normality and adjustment for multiple comparisons
☐	☒	A full description of the statistical parameters including central tendency (e.g. means) or other basic estimates (e.g. regression coefficient) AND variation (e.g. standard deviation) or associated estimates of uncertainty (e.g. confidence intervals)
☐	☒	For null hypothesis testing, the test statistic (e.g. F, t, r) with confidence intervals, effect sizes, degrees of freedom and P value noted<br>*Give P values as exact values whenever suitable.*
☒	☐	For Bayesian analysis, information on the choice of priors and Markov chain Monte Carlo settings
☒	☐	For hierarchical and complex designs, identification of the appropriate level for tests and full reporting of outcomes
☒	☐	Estimates of effect sizes (e.g. Cohen's d, Pearson's r), indicating how they were calculated

Our web collection on statistics for biologists contains articles on many of the points above.

Software and code

Policy information about availability of computer code

Data collection	We used MATLAB (version 2017b, Mathworks) custom scripts, with functions provided by the Vantage 4.0.0 system (Verasonics), to acquire ultrasound images. Phase contrast microscopy images were acquired using Zen 2 Core (version 2.5, Zeiss) software. Optical Density of samples was measured using the NanoDrop 2000c software (version 1.5, Thermo Fisher Scientific). All custom code will be available on the Shapiro Lab GitHub (https://github.com/shapiro-lab) upon publication.
Data analysis	We used MATLAB (2021a or 2019a, Mathworks), Python (version 3.7.12; packages Pandas, Numpy, and Seaborn), ImageJ (version 1.53c, NIH) and Prism (version 9, Graphpad) for data and image analysis and plotting. FlowJo (version 10.8.0) was used to process flow cytometry data. ClustalW (1.2.4) was used through the EMBL-EBI web interface (https://www.ebi.ac.uk/Tools/services/web_clustalo/toolform.ebi) to produce multiple sequence alignments. Illustrations were made in Affinity Designer (version 1.10.0, Serif Europe) and with BioRender.com.

For manuscripts utilizing custom algorithms or software that are central to the research but not yet described in published literature, software must be made available to editors and reviewers. We strongly encourage code deposition in a community repository (e.g. GitHub). See the Nature Portfolio guidelines for submitting code & software for further information.

Data

Policy information about availability of data

All manuscripts must include a data availability statement. This statement should provide the following information, where applicable:

- Accession codes, unique identifiers, or web links for publicly available datasets
- A description of any restrictions on data availability
- For clinical datasets or third party data, please ensure that the statement adheres to our policy

Plasmids will be made available through Addgene upon publication. Raw data used for plotting is provided as Source Data. All other materials and data are available from the corresponding author upon reasonable request.

Field-specific reporting

Please select the one below that is the best fit for your research. If you are not sure, read the appropriate sections before making your selection.

☒ Life sciences ☐ Behavioural & social sciences ☐ Ecological, evolutionary & environmental sciences

For a reference copy of the document with all sections, see nature.com/documents/nr-reporting-summary-flat.pdf

Life sciences study design

All studies must disclose on these points even when the disclosure is negative.

Sample size	The numbers of biological and technical replicates were chosen based on preliminary experiments, so as to provide sufficient power for statistical comparison.
Data exclusions	Induced liquid cultures of E. coli Nissle with the pBAD-bARGSer-AxeTxe plasmid were screened with phase contrast microscopy to verify the presence of gas vesicles before being imaged with ultrasound because certain lots of LB media resulted in no or minimal gas vesicle expression, likely due to variations in the batches of tryptone. Cultures that exhibited no or minimal gas vesicle expression were excluded.
Replication	Replicates are reported in the figure legends.
Randomization	Animals were randomly distributed into cages and ear-punched by animal care staff. Cages of animals were randomly chosen for GV-expressing versus control (uninduced or fluorescent-protein-expressing) conditions. In all other experiments, samples were allocated randomly.
Blinding	Blinding was not applicable to our study because our experiments did not involve human participants and all data collection, processing, and analysis methods were quantitative and identical across experimental groups.

Reporting for specific materials, systems and methods

We require information from authors about some types of materials, experimental systems and methods used in many studies. Here, indicate whether each material, system or method listed is relevant to your study. If you are not sure if a list item applies to your research, read the appropriate section before selecting a response.

Materials & experimental systems

n/a	Involved in the study	
☐	☒	Antibodies
☐	☒	Eukaryotic cell lines
☒	☐	Palaeontology and archaeology
☐	☒	Animals and other organisms
☒	☐	Human research participants
☒	☐	Clinical data
☒	☐	Dual use research of concern

Methods

n/a	Involved in the study	
☒	☐	ChIP-seq
☐	☒	Flow cytometry
☒	☐	MRI-based neuroimaging

Antibodies

Antibodies used	For anti-E. coli staining of tumor sections, polyclonal rabbit anti-E. coli antibody (Virostat; catalogue number 1001) and the Opal Polymer anti-Rabbit HRP Kit (Akoya Biosciences; catalogue number ARR1001KT) were used.
Validation	Virostat: https://www.virostat-inc.com/products1/e-coli-1, Akoya: https://www.akoyabio.com/phenoptics/opal-kits-reagents/opal-secondary-antibodies/

Eukaryotic cell lines

Policy information about cell lines

Cell line source(s)	HEK293T, MC26, 3T3, Huh7 and MDA-MB-231 cells were ordered from American Type Culture Collection (ATCC).
Authentication	The cells were authenticated by ATCC before delivery using short tandem repeat (STR) profiling.
Mycoplasma contamination	The cells were certified not contaminated by ATCC and not tested for mycoplasma contamination subsequently.
Commonly misidentified lines (See ICLAC register)	No commonly misidentified cell lines were used in this study.

Animals and other organisms

Policy information about studies involving animals; ARRIVE guidelines recommended for reporting animal research

Laboratory animals	Female BALB/cJ mice aged 6-8 weeks and female NSG mice aged 12-14 weeks were used for in vivo experiments. Animal housing room temperatures are monitored at all times and maintained between 71 and 75 degrees F for most species according to their physiological needs. Humidity is maintained between 30-70%. Light intensity and light cycle timing are carefully regulated and monitored in Caltech laboratory animal facilities. Automated light timers ensure a consistent light-dark cycle with 13 hours on and 11 hours off.
Wild animals	This study did not involve wild animals.
Field-collected samples	This study did not involve samples collected from the field.
Ethics oversight	Institutional Animal Care and Use Committee (IACUC) of the California Institute of Technology (Caltech) for animal experiments.

Note that full information on the approval of the study protocol must also be provided in the manuscript.

Flow Cytometry

Plots

Confirm that:

☒ The axis labels state the marker and fluorochrome used (e.g. CD4-FITC).

☒ The axis scales are clearly visible. Include numbers along axes only for bottom left plot of group (a 'group' is an analysis of identical markers).

☒ All plots are contour plots with outliers or pseudocolor plots.

☒ A numerical value for number of cells or percentage (with statistics) is provided.

Methodology

Sample preparation	MDA-MB-231 cells were trypsinized and resuspended in excess DMEM + 10%FBS. Cells were then centrifuged at 300xg for 6 minutes and media was removed and replaced with DMEM + 1%FBS for sorting. Cells were finally filtered through blue cap 5mL cell strainers. For Post-sort flow analysis the procedure was same, except PBS was used as final resuspension buffer.
Instrument	FACS Aria SORP, FACS Aria Fusion and MACSQuant VYB analyzer
Software	FlowJo
Cell population abundance	Post-sort flow cytometry analysis performed after outgrowth and reinduction of sorted cells. Purity was determined as the fraction of single cells that fall within the double-positive gate (95.1%). See Figure S16d.
Gating strategy	Gating strategy is outlined in figure S16d. Prior to the first plot, cells were gated for FSC-SSC size and Width/Area doublet discrimination.

☒ Tick this box to confirm that a figure exemplifying the gating strategy is provided in the Supplementary Information.
