# Supplementary figures and images for: Genomically mined acoustic reporter genes for real-time in vivo monitoring of tumors and tumor-homing bacteria
Source: Nat Biotechnol. 2023 Jan 2;41(7):919–31. doi: 10.1038/s41587-022-01581-y (PMC10344784; doi:10.1038/s41587-022-01581-y)

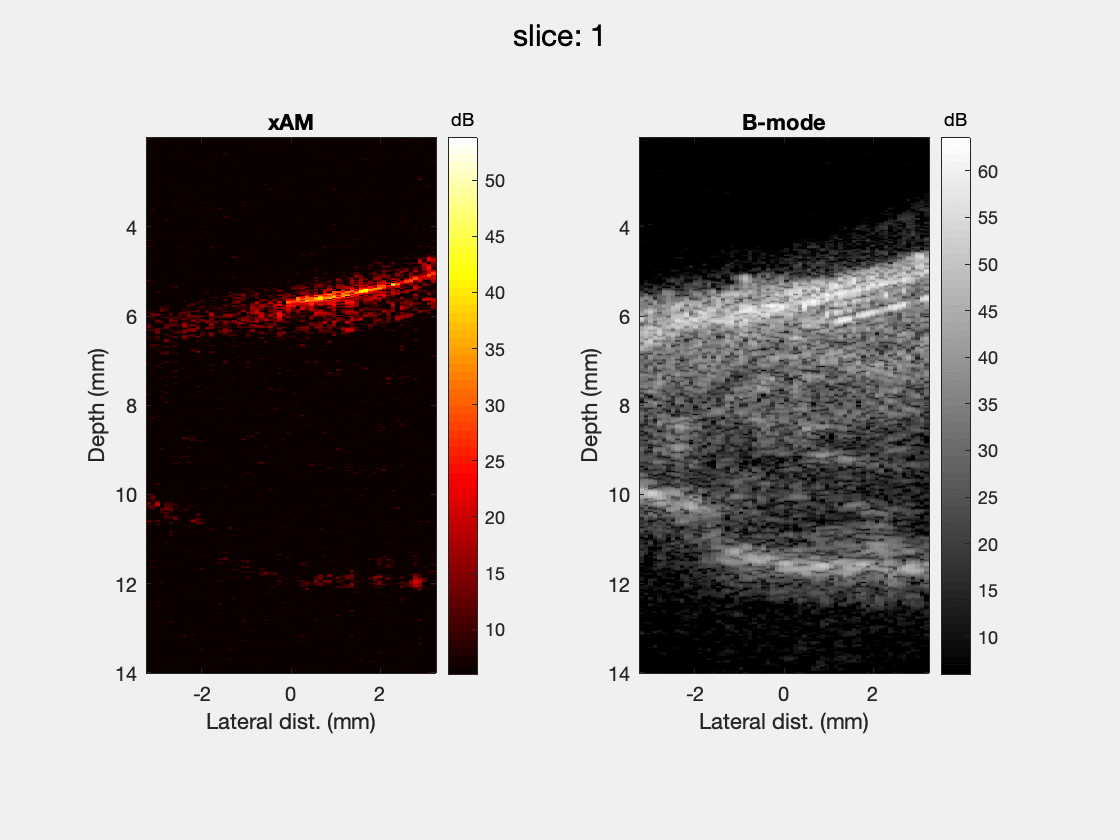

Supplement: Supplementary file 4 — Supplementary Video 1: xAM/B-mode tomogram of an induced orthotopic tumor imaged on day 12. A representative tomogram of an orthotopic MDA-MB-231-mARGAna tumor imaged after 12 days of doxycycline induction. Each slice in the tomogram is separated by 100 µm. [file 41587_2022_1581_MOESM4_ESM.gif]

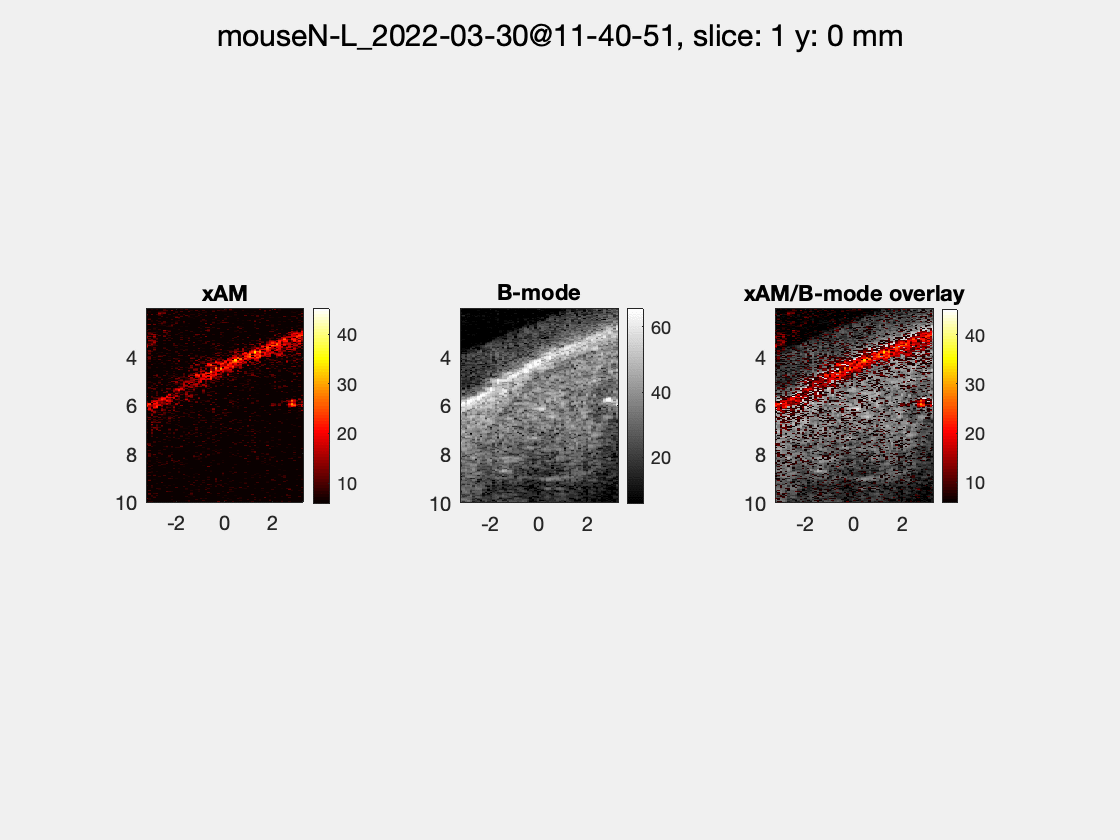

Supplement: Supplementary file 5 — Supplementary Video 2: xAM/B-mode tomogram of an induced chimeric tumor imaged on day 5. A representative tomogram of a chimeric MDA-MB-231-mARGAna tumor imaged after 5 days of doxycycline induction. Each slice in the tomogram is separated by 200 µm. [file 41587_2022_1581_MOESM5_ESM.gif]

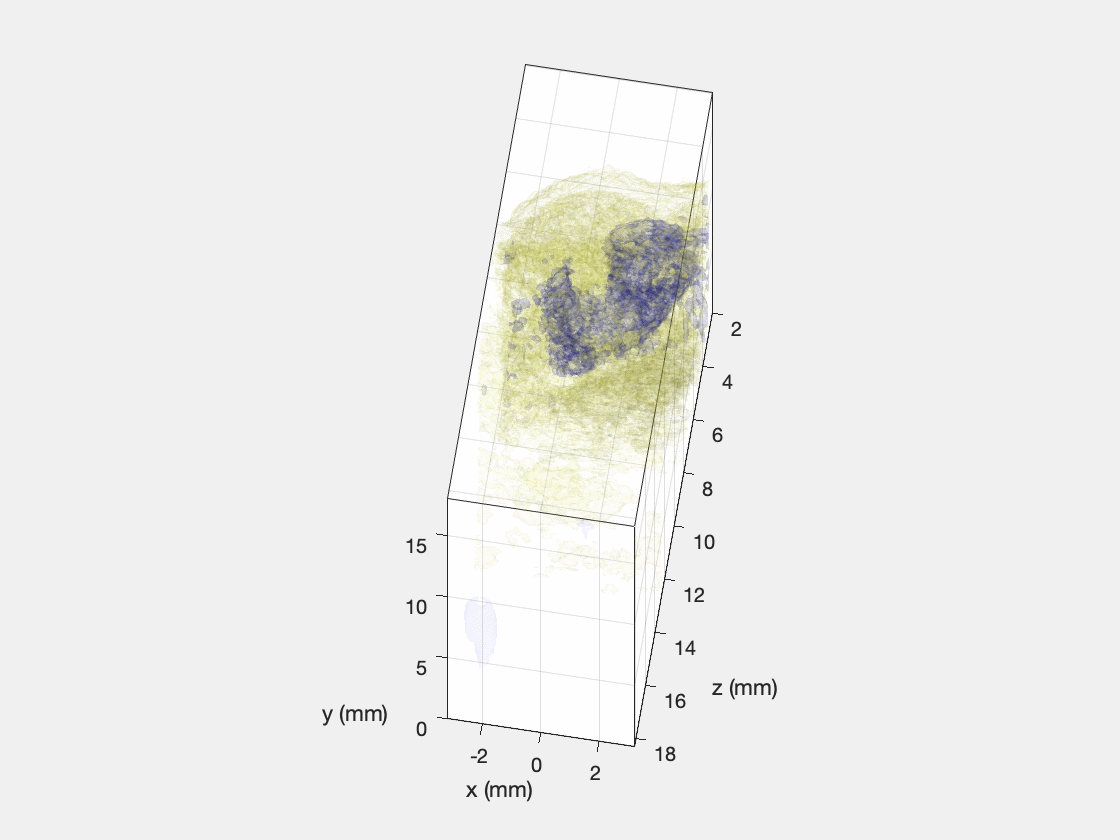

Supplement: Supplementary file 6 — Supplementary Video 3: xAM/B-mode 3D reconstruction of an induced chimeric tumor imaged on day 5. 3D B-mode and xAM data were smoothened and converted to isosurfaces using MATLAB. Yellow 3D map represents B-mode density; blue 3D map represents xAM density. [file 41587_2022_1581_MOESM6_ESM.gif]

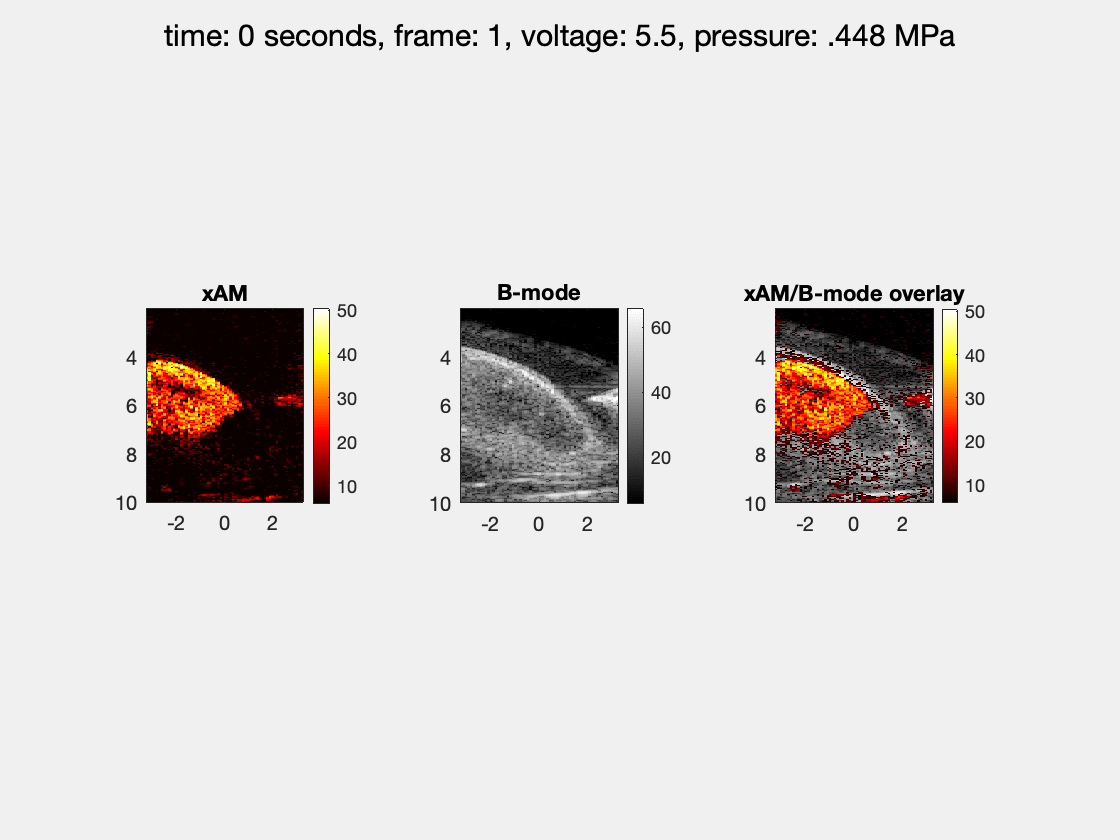

Supplement: Supplementary file 7 — Supplementary Video 4: Representative xAM/B-mode video of a chimeric tumor biopsy procedure sampling the xAM-positive region. [file 41587_2022_1581_MOESM7_ESM.gif]

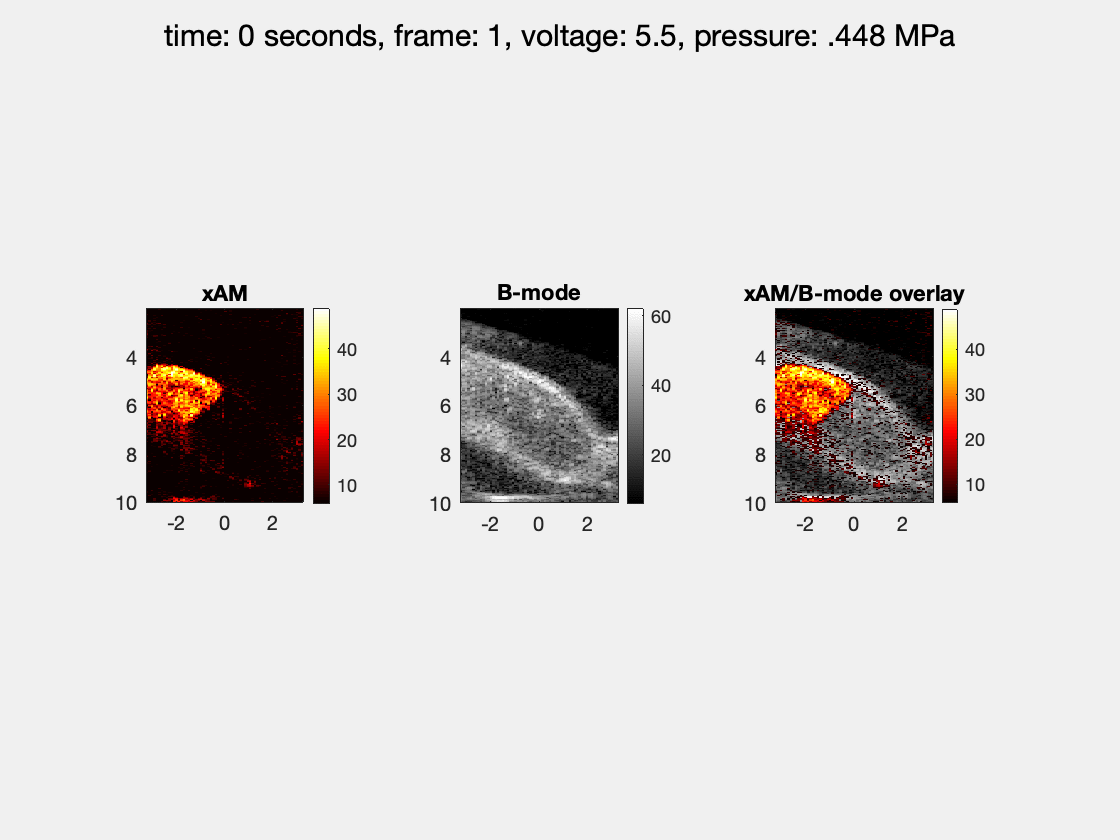

Supplement: Supplementary file 8 — Supplementary Video 5: Representative xAM/B-mode video of a chimeric tumor biopsy procedure sampling the xAM-negative region. [file 41587_2022_1581_MOESM8_ESM.gif]
